# Salidroside Ameliorates Alzheimer's Disease by Targeting NLRP3 Inflammasome-Mediated Pyroptosis

**DOI:** 10.3389/fnagi.2021.809433

**Published:** 2022-01-21

**Authors:** Yawen Cai, Yuhui Chai, Yu Fu, Yingdi Wang, Yiming Zhang, Xue Zhang, Lingpeng Zhu, Mingxing Miao, Tianhua Yan

**Affiliations:** ^1^Department of Physiology and Pharmacology, School of Basic Medicine and Clinical Pharmacy, China Pharmaceutical University, Nanjing, China; ^2^Center of Clinical Research, The Affiliated Wuxi People's Hospital of Nanjing Medical University, Wuxi, China; ^3^Center of National Pharmaceutical Experimental Teaching Demonstration, China Pharmaceutical University, Nanjing, China

**Keywords:** Alzheimer's disease, salidroside, pyroptosis, NLRP3 inflammasome, TLR4

## Abstract

Amyloid β-protein (Aβ) is reported to activate NLRP3 inflammasomes and drive pyroptosis, which is subsequently involved in the pathogenesis of neurodegenerative diseases, such as Alzheimer's disease (AD). To date, the pathogenesis of AD is unfortunately insufficiently elucidated. Therefore, this study was conducted to explore whether Salidroside (Sal) treatment could benefit AD by improving pyroptosis. Firstly, two animal models of AD, induced, respectively, by Aβ1-42 and D-galactose (D-gal)/AlCl_3_, have been created to assist our appreciation of AD pathophysiology. We then confirmed that pyroptosis is related to the pathogenesis of AD, and Sal can slow the progression of AD by inhibiting pyroptosis. Subsequently, we established the D-gal and Nigericin-induced PC12 cells injury model *in vitro* to verify Sal blocks pyroptosis mainly by targeting the NLRP3 inflammasome. For *in vivo* studies, we observed that Aβ accumulation, Tau hyperphosphorylation, neurons of hippocampal damage, and cognitive dysfunction in AD mice, caused by bilateral injection of Aβ1-42 into the hippocampus and treatments with D-gal combine AlCl_3_. Besides, accumulated Aβ promotes NLRP3 inflammasome activation, which leads to the activation and release of a pro-inflammatory cytokine, interleukin-1 beta (IL-1β). Notably, both Aβ accumulation and hyperphosphorylation of Tau decreased and inhibited pyroptosis by downregulating the expression of IL-1β and IL-18, which can be attributed to the treatment of Sal. We further found that Sal can reverse the increased protein expression of TLR4, MyD88, NF-κB, P-NF-κB, NLRP3, ASC, cleaved Caspase-1, cleaved GSDMD, IL-1β, and IL-18 *in vitro*. The underlying mechanism may be through inhibiting TLR4/NF-κB/NLRP3/Caspase-1 signaling pathway. Our study highlights the importance of NLRP3 inflammasome-mediated pyroptosis in AD, and how the administration of pharmacological doses of Sal can inhibit NLRP3 inflammasome-mediated pyroptosis and ameliorate AD. Thus, we conclude that NLRP3 inflammasome-mediated pyroptosis plays a significant role in AD and Sal could be a therapeutic drug for AD.

## Introduction

Alzheimer's disease (AD) is an irreversible neurodegenerative disorder characterized by memory loss and language deterioration, and its incidence rate ranks first globally (Alzheimer's Association, 2021). The World Alzheimer Report 2021 reported that 50 million people worldwide have dementia. Every 3 s, someone in the world develops dementia. It is estimated that those suffering from dementia will grow from 55 to 139 million people during the period 2019–2050 (World Alzheimer Report 2021, 2021).

β-Amyloid (Aβ) deposits, Tau proteins hyperphosphorylated, and neuroinflammation are neuropathological hallmarks in AD (Emre et al., 2021). According to the hypothesis of Aβ, on the one hand, the aggregation of Aβ can initiate a cascade of reactions, including triggering hyperphosphorylation of Tau to form neurofibrillary tangles (NFTs), neuroinflammation, and neuronal degeneration, contributing to the onset of AD (Zhou and Fukushima, 2020). On the other hand, the activation and proliferation of astrocytes and microglia caused by Aβ accumulation (Terrill-Usery et al., 2014) leads to the continuous exposure of neurons to pro-inflammatory mediators and results in neuronal dysfunction or death (Heneka et al., 2014). Thus, the vital pathological features of AD outlined above can cause different degrees of neuronal loss and brain atrophy (Zhuang et al., 2019). Therefore, death neuronal is a root reason for AD and other neurodegenerative diseases (Hambright et al., 2017). Furthermore, emerging evidence indicates pyroptosis may serve as the predominant form of neuronal death in AD following apoptosis, autophagy, and necrosis (Fricker et al., 2018; Shen et al., 2021).

Pyroptosis is a programmed cell death mediated by Gasdermin D (GSDMD) (Shi et al., 2017), characterized by swelling, rupture of the cell membrane, drive to release pro-inflammatory cytokines, and intracellular contents, and then triggering the inflammatory response (Yu et al., 2021). Pyroptosis occurs when activated caspase-1 or caspase-4/5/11 cleaves the protein GSDMD, releasing the GSDMD-N subunit to form cell membrane pores (Kovacs and Miao, 2017), which has been tightly linked to AD pathogenesis. Suppressed pyroptosis may be a protective measure for neurons (Zhao et al., 2019). Studies have suggested that the inflammasome was involved in the onset of AD through triggering pyroptosis (Dempsey et al., 2017). Among all inflammasomes, including nod-like receptor protein 1 (NLRP1), nod-like receptor protein 3 (NLRP3), the nuclear effector protein 4 (NLRC4), and absent in melanoma 2 (AIM2), extensive studies have focused on the NLRP3 inflammasome.

NLRP3 inflammasome comprises three main components: receptor protein NLRP3, the adaptor protein apoptosis-associated speck-like protein (ASC), and the effector protein caspase-1 (Rathinam and Fitzgerald, 2016). It is initiated by a variety of stimuli, namely, microbial infection PAMPs (such as viral DNA, microbes, and the components of the bacterial cell wall) and DAMPs (such as adenosine triphosphate (ATP), uric acid (UA) crystals, Aluminum adjuvant, and Aβ peptide) (Huang et al., 2021). Numerous research studies have examined how NLRP3 inflammasome is involved in AD pathogenesis through meditating pyroptosis. A study by Daniels et al. (2016) demonstrated that Fenamate NSAIDs improved the microglia-mediated neuroinflammation and memory loss in 3×TgAD mice by indirectly inhibiting the activation of NLRP3 inflammasome. Then, Chiu et al. (2021) found that SG-Tang could act as a neuroprotective agent against Aβ aggregation and neuroinflammation by regulating the NLRP1/NLRP3 pathway. Subsequently, Kuwar et al. (2021) suggested that loaded Aβ can be reduced significantly, alleviate neuroinflammation, improve hippocampal neuronal activity, and relieve cognitive function in APP/PS1 mice via inhibiting NLRP3 inflammasome with JC124. Even though limited studies have probed the role and underlying mechanism of NLRP3 inflammasome-mediated pyroptosis in AD, the mechanisms of how NLRP3 inflammasome drive pyroptosis need further study.

So far, clinical treatment of AD therapeutics has been dominated by the use of western medicine, that is to say, acetylcholinesterase (AChE) inhibitors, NMDA receptor (NMDAR) antagonists, amyloid production or secretion inhibitors, and inflammation inhibitors (Orlov, 2019). Nevertheless, these drugs only partially relieve symptoms for a short time but cannot delay the progression of AD. To our relief, recent studies have reported that phytopharmaceuticals, Salidroside can improve neuron impairments and cognitive deficits in AD (Xie et al., 2020).

*Rhodiola rosea* L., a small genus of the Crassulaceae family, has a long history of wide use as a botanical medicine in Europe, Asia, and the US (Zhong et al., 2018). With the continuous development and improvement of scientific research technology, many studies have examined the chemical constituents of Rhodiola plants at home and abroad. The main pharmacological active ingredients isolated from various Rhodiola are salidroside and its aglycone (tyrosol), rosavin or rosavidine, pyridine, and rhodiosin and rhodionin, etc (Zhang et al., 2021). Among them, Salidroside is widely studied. Sal (2-(4-hydroxyphenyl) ethyl-β-D-glucopyranoside) (**Figure 2A**) is a phenolic glycoside compound that is the main active ingredient extracted from Rhodiola Rose (Fan et al., 2021). It has potent pharmacological activities, mainly for preventing or treating disorders like neurodegenerative diseases, cerebral ischemia, fatigue, hypoxia, diabetes, and cancer (Grech-Baran et al., 2015). Furthermore, studies have demonstrated that Sal can effectively reduce Aβ1-42 deposition and neuroinflammation in the brains of AD mice (Xie et al., 2020), improve cognitive decline during aging, and thus exert neuronal protective effects (Jin et al., 2016). In their study, Xing et al. (2020) found that Sal attenuated pyroptosis by inhibiting caspase-1 activation, GSDMD cleaving, and reducing the interleukin-1β (IL-1β) release, ultimately reducing the form of atherosclerotic plaque. In addition, in PD-related studies Zhang X. et al. (2020) also demonstrated that Sal has a therapeutic effect on PD by inhibiting pyroptosis and thus dopamine neuron death. All in all, the above studies consistently conclude that Sal plays a potential neuroprotective role by inhibiting pyroptosis. Regrettably, the mechanism of how Sal protects neurons in AD through inhibiting pyroptosis is incomplete and has not been clarified to date.

This study aimed to investigate the interaction between AD and NLRP3 inflammasome-mediated pyroptosis via established Aβ1-42, D-gal/AlCl_3_ mouse models *in vivo*, and a D-gal, Nigericin treated PC12 cells damage model *in vitro*.

## Materials and Methods

### Chemicals

Salidroside was provided by Meilunbio (Dalian, China, purity > 98%). Aβ1-42 (Amyloid Beta 1-42) was obtained from Sigma-Aldrich Inc. (St. Louis, MO, United States). D-galactose, AlCl_3_, and Donepezil Hydrochloride were obtained from Aladdin (Shanghai, China). Fetal bovine serum was purchased in Thermo Fisher Scientific (WLM, Mass, United States). Enzyme-linked immunosorbent assay (ELISA) kits of Aβ1-42, interleukin (IL)-18, and IL-1β were purchased from Elabscience (Wuhan, China). Antibodies against P-Tau, NLRP3 were obtained from Abcam (Cambridge, United Kingdom). Cleaved Caspase-1 was purchased in Thermo Fisher Scientific (WLM, Mass, United States). The primary antibodies against MyD88, p-NF-κB, NF-κB, cleaved GSDMD, and β-actin were purchased from Cell Signaling Technology (Danvers, MA, USA). Aβ, Tau, ASC, IL-1β, and IL-18 were provided by Proteintech (Chicago, USA), and the anti-TLR4 antibody was purchased from Santa Cruz Biotechnology (Santa Cruz, CA). All chemicals and reagents are summarized in Supplementary Table S1. All antibodies are summarized in Supplementary Table S2.

### Animals

The present study uses male C57BL/6J mice (8 weeks old) with a bodyweight of 18–22 g, provided by the Nanjing Qinglongshan Animal Cultivation Farm (Nanjing, China). Animals were raised in an environment with constant temperatures (25 ± 2°C), light/dark cycles (12/12 h), and relative humidity (60 ± 10%). During this period, the animal has free access to food and water. Animals were provided with standard feeding and allowed water and libitum.

All the animal procedures were performed according to the 3 R's principle (Replacement, Reduction, and Refinement) and the Guide for Care and Use of Laboratory Animals (Bethesda, MD, United States). The experimental protocols were approved by the Animal Ethics Committee of the China Pharmaceutical University. All efforts were made to minimize the number of animals used and the distress suffered.

### Preparation of the Aβ1-42 Oligomer

The Aβ1-42 peptide was treated as previously described (Xu et al., 2021) to obtain Aβ oligomers. Briefly, HFIP-treated Aβ1-42 was resuspended in dimethylsulfoxide (DMSO) when evaporating the HFIP to form a peptide film. Then, Aβ1-42 was incubated at 37°C for 7 days, and stored at −80°C (Xiao et al., 2020).

### Establishment of the AD Mice Model

**Figure 1:**
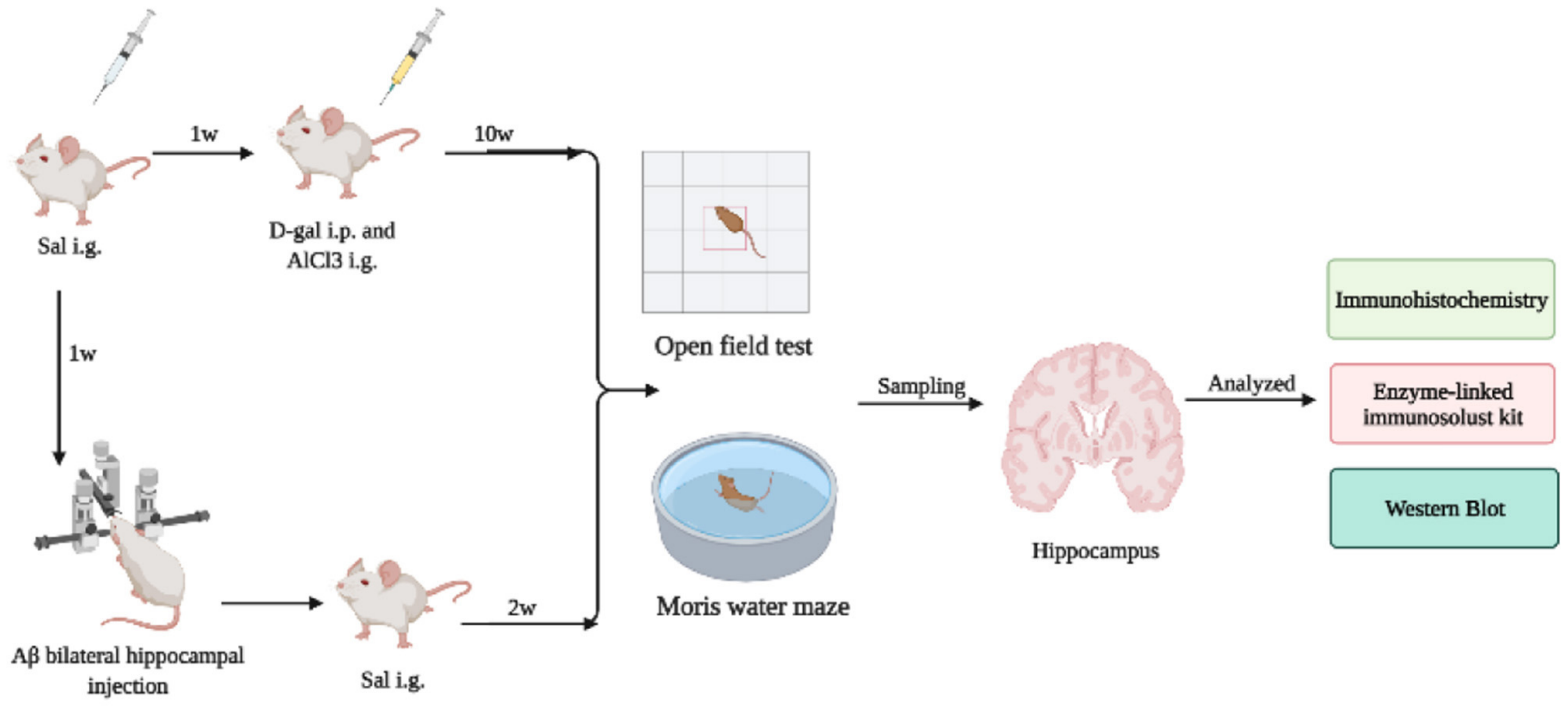
The study flow diagram for animal experiments. The experimental animal design was mainly divided into two parts. Part I investigated the influence of Sal on the AD mice model induced by Aβ1-42. Part II further explored the potential mechanism of Sal on D-gal/AlCl_3_-induced AD mice and inquired further as to how the Sal mechanism acts in pyroptosis. Firstly, an Open-field test and Morris water maze were performed to test the behavioral performance of AD mice. Then, mice were killed by cervical dislocation to collect hippocampal tissues and collected for further histologic analysis, including Immunohistochemical staining, Enzyme Immunoassay Kit, and Western blotting analysis.

The study flow diagram for animal experiments is shown in Figure 1. The experimental animal design was mainly divided into two parts. Part I is to investigate the influence of Sal on the AD mice model induced by Aβ1-42. Part II is to further explore the potential mechanism of Sal on D-gal/AlCl_3_-induced AD mice and to inquire about the Sal action mechanism in pyroptosis. Firstly, an Open-field test and Morris water maze were performed to test the behavioral performance of AD mice. Then, mice were killed by cervical dislocation to collect hippocampal tissues and collected for further histologic analysis, including Immunohistochemical staining, Enzyme Immunoassay Kit, and Western blotting analysis.

#### Aβ1-42 Mice Model

As reviewed in other studies (Fu et al., 2019; Sun et al., 2020b), forty-eight male C57BL/6J mice were randomized into six groups (*n* = 8): (1) control (DMSO Saline), (2) AD model (Aβ1-42), (3) positive control (Aβ1-42+Donepezil (DNP), 1 mg/kg), (4) low-dose (Aβ1-42+Sal, 20 mg/kg), (5) middle-dose (Aβ1-42+Sal, 40 mg/kg), and (6) high-dose (Aβ1-42+Sal, 80 mg/kg) groups.

Mice were set on a stereotaxic apparatus after being anesthetized with pentobarbital sodium (50 mg/kg, i.p.), and located in: Bregma = −2 mm, Sagittal suture = 1.5 mm, and Skull = −2 mm. The dura was exposed with the use of a skull drill to drill. Subsequently, the micro-syringe was inserted into the left and right hippocampus to inject Aβ1-42 Oligomer, inject 5 μl on each side for 5 min, and keep 2 min to prevent reflux.

#### D-gal and AlCl_3_ Mice Model

According to relevant literature (An et al., 2017; Liang et al., 2018), the mice were randomly divided into six groups (*n* = 10): (1) control (Saline + Pure water); (2) D-gal (150 mg/kg)/AlCl_3_(20 mg/kg); (3) D-gal/AlCl_3_+DNP (1 mg/kg); (4) D-gal/AlCl_3_+ Sal (20 mg/kg); (5) D-gal/AlCl_3_+ Sal (40 mg/kg); (6) D-gal/AlCl_3_+ Sal (80 mg/kg).

After treatment with Sal and DNP for 7 days, D-gal (intraperitoneal injection, 150 mg/kg/d) and AlCl_3_ (intragastric administration, 20 mg/kg/d) were given in the experimental group. By contrast, the Sal and DNP group were given not only D-gal/AlCl_3_ but also treatment with Sal or DNP. The control group was given pure water (intragastric administration) and intraperitoneal injection of Saline daily. Subsequently, the Open-field test was used to test autonomous activities and exploration capabilities, and the Morris water maze evaluated the spatial learning and memory ability after administration for 10 weeks (Wang et al., 2019).

### Behavioral Tests

#### Open-Field Test

The open-field test is mainly used to exclude animal movement obstacles, detect animal autonomous activities and exploration capabilities (Kraeuter et al., 2019). The open-field test box comprises white wooden boards (40 × 40 × 45 cm) divided into central and peripheral areas. The OFT was carried out in a quiet environment, as described by Sun et al. (2020a). All mice were adapted to the new experimental environment for 30 min before the test began. The total distance and time traveled in the open field, and the total number of crossings in the central square were recorded within 5 min. After each test, the feces was cleaned up using 75% alcohol to eliminate any odor and avoid interfering with the exploration activities of other mice.

#### Morris Water Maze Test

The Morris water maze test was performed to assess learning and memory ability (Zhang et al., 2018). In this study, the MWM was conducted in mice, as described by Wang W. Z. et al. (2021). Mice were placed in a circular pool (120 cm diameter, 40 cm height) filled with milky white water at room temperature (25°C) with the escape platform (8 cm diameter) submerged ~1 cm below the surface. A video-tracking system recorded the animals' movement. Mice were trained four times per day over five consecutive days, and each trial lasted for 90 s.

In the place navigation test, mice were manually guided to the platform stay continued 15 s, when they failed to search the platform in persistent 90 s. The phrase escape latency describes the time it took the mice to find the platform by themselves and stay there for over 15 s. On the sixth day of the MWM, the spatial probe test was conducted as follows. First, the mice were placed in the quadrant opposite the target quadrant without the platform. Then, the number of times they crossed the platform in the target quadrant within 90 s, travel time and distance in the target quadrant, and the route map of the search for the platform were recorded. Finally, data were analyzed by one-way ANONY.

### Nissl Staining

Nissl staining was also performed as previously described (Zhu et al., 2020). First, the sections were dehydrated through graded ethanol solutions (75, 85, 95, and 100%) and stained with 0.5% cresyl violet (Beyotime, China). Next, excess cresyl violet was removed with PBS three times for 5 min then the slice was quickly dehydrated in 100% ethanol for 10 s. They were then covered with a mounting medium. Finally, the slices were imaged using a light microscope (Nikon, Ts2R, Japan), and Image J software was used to analyze them.

### Cell Culture

Rat pheochromocytoma PC12 cells were purchased by the Chinese Academy Cell Resource Center (Shanghai, China). The cells were grown in a medium containing 10% fetal bovine serum (FBS), 100 U/mL penicillin, and 100 μg/mL streptomycin in an incubator (Thermo Scientific) under 37°C, 5% CO_2_. The medium was replaced every 2–3 days. Cells under the logarithmic phase of culture were used for experiments. Furthermore, the cells were planted at a specified density based on each experiment.

### Cell Treatment

PC12 cells (2 × 10^∧^4 cells/well) were seeded into 6-well plates and cultured for 24 h. Next, the PC12 cells were pre-treated with Sal (2, 10, 50 μM) for 2 h and then stimulated with D-gal (15 mg/ml) for 48 h. At last, all the cells were collected for analyses.

PC12 cells (2 × 10^∧^4 cells/well) were planted into 6-well plates and cultured for 24 h. Next, the PC12 cells were pre-treated with NLRP3 agonist Nigericin (10 μM) or Sal (50 μM) for 2 h and then treated with D-gal (15 mg/ml) for 48 h at 37°C, 5% CO_2_ incubator. Finally, the cells in all groups were collected for subsequent analyses.

### Cell Viability Assay

PC12 cells treatments were conducted in the 96-well plates, following Wang Y. et al. (2021). Briefly, PC12 cells were planted into 96-well plates at a density of 1 × 10^∧^4 cells per well and incubated with D-gal (15 mg/ml) for 48 h in the presence or absence of Sal (2, 10, 50 μM), followed by the addition of CCK-8 solution (Beyotime Biotechnology, Nantong, China) and incubated for 1 h. Ultimately, the OD values of the cells at 450 nm were monitored using a microplate reader, and the cell viability was calculated by the formula as follows: Cell viability (%) = (OD [experiment]-OD [blank])/ (OD [control]-OD [blank]) × 100%.

### SiRNA Transfection and Cell Viability Assay

PC12 cells were transfected with a specific NLRP3 siRNA (100 nM) or non-binding control siRNA (100 nM) using a siRNA kit (RiboBio, Guangzhou, China) according to the manufacturer's instructions. After transfection for 24 h, the cells were treated with 50 μM Sal for 24 h and exposed to D-gal or not. The cells were collected for Western blotting analysis. The cell viability was analyzed using a CCK-8 solution (Beyotime Biotechnology, Nantong, China). The experiment details were performed following the manufacturer's instructions. All siNLRP3 sequences are summarized in Supplementary Table S3.

### Immunohistochemistry Staining

Immunohistochemistry (IHC) analysis was performed as described in previous reports (Chou and Yang, 2021), with minor modifications. We collected the hippocampus of AD mice to investigate the action of Sal on the protein of Aβ1-42, inflammatory-associated proteins (TLR4/NF-κB), and pyroptosis-associated proteins (NLRP3/Caspase-1). Briefly, the whole brains were dehydrated, followed by immersion in paraffin, and subsequently, slices were cut. The sections were dewaxed by xylene and hydrated in graded ethanol, then micro-waved in sodium citrate buffer. Next, 3% hydrogen peroxide was applied to block the endogenous peroxidase for 10 min. Then the slices were incubated with 5% fetal bovine serum for 1 h at room temperature, before being treated with the primary antibodies Aβ1-42 (Proteintech, 25524-1-AP, 1: 400), p-Tau (Abcam, ab109390, 1: 200), NLRP3 (Abcam, ab263899, 1: 200), caspase-1 (Thermo Fisher Scientific, PA5-99390, 1: 200), and TLR4 (Santa Cruz, sc-293072, 1: 50) at 4°C overnight. The sections were washed and incubated on the second day with the goat anti-rabbit IgG (the first four primary antibodies) and the goat anti-mouse IgG (the last primary antibodies) secondary antibodies for 30 min. DAB chromogen reagent was used to develop color with the slices. Lastly, we collected the images with an inverted fluorescent microscope (Nikon, Ts2R, Japan), and Image J quantified the image.

### Immunofluorescence Staining

The immunofluorescence was performed with some modifications, following a previous description by Zhao et al. (2021). In short, after Immunol Staining Fix Solution was applied to fix the PC12 cells for 30 min it was washed in PBS. Then 0.2% Triton X-100 (9002-93-1, Sigma, USA) was added for 10 min. After being washed with PBS, cells were blocked with 5% BSA for 1 h. Subsequently, the slices were incubated with rabbit anti-caspase-1 antibody (1: 400, PA5-99390, Invitrogen, USA), rabbit anti-NLRP3 antibody (1: 200, ab263899, Abcam, USA), and mouse anti-TLR4 (Santa Cruz, sc-293072, 1:50), overnight at 4°C. On the second day, PBS washed the slides and then incubated them with the secondary antibody (1:400) corresponding to the primary antibody in a dark room for 2 h at room temperature. PBS was washed again, and the 4′,6-diamidino-2-phenylindole (DAPI, (Thermo Fisher, WLM, Mass, United States) was applied to stain the cells for 5 min at room temperature. Finally, the image was photographed by an inverted fluorescence microscope (Nikon, Ts2R, Japan) and analyzed by Image J.

### Enzyme-Linked Immunosorbent Assay

The enzyme-linked immunosorbent assay (ELISA) kits were applied to determine the concentration of Aβ1-42, IL-1β, and IL-18 in mouse brain and IL-1β, IL-18 in PC12 cell supernatant, following the manufacturer's instructions (Elabscience, China).

### Western Blot Analysis

The hippocampus tissue of AD mice was homogenated in RIPA buffer containing phenylmethylsulfonyl fluoride (PMSF). Meanwhile, PC12 cells were lysed with lysis buffer containing protease inhibitors. Firstly, the samples were centrifuged at 12,000 rpm/15 min at 4°C. In turn, the protein content was detected by the BCA protein assay kit (Beyotime Biotechnology, Nantong, China). Then, Western blotting was performed.

As mentioned by Shi et al. (2021), the procedures were as follows: protein samples were separated by electrophoresis using 12% SDS polyacrylamide gel electrophoresis and transferred onto PVDF membranes. Membranes were hindered with 5 % skim milk and incubated with primary antibodies Aβ1-42 (Proteintech, 25524-1-AP, 1:1,000), p-Tau (Abcam, ab109390, 1:3,000), Tau (Proteintech, 66499-1-Ig, 1:1,000), TLR4 (Santa Cruz, sc-293072, 1:200), MyD88 (Cell Signaling Technology, #4283, 1:1,000), NF-κB (Cell Signaling Technology, #8242, 1:1,000), p-NF-κB (Cell Signaling Technology, #3033, 1:1,000), NLRP3 (Abcam, ab263899, 1:1,000), ASC (Proteintech, 10500-1-AP, 1:1,000), cleaved Caspase-1 (Invitrogen, PA5-99390, 1:1,000), Cleaved Gasdermin D (Cell Signaling Technology, #10137, 1:1,000), IL-1β (Proteintech, 16806-1-AP, 1:1,000), IL-18 (Proteintech, 10663-1-AP, 1:1,000), and β-actin (Cell Signaling Technology, #3700, 1:1,000) overnight at 4°C. The membranes were then incubated with corresponding second antibodies at room temperature for 1 h after TBST wash 3 times. Finally, an ECL kit was used to image the gel imaging system to detect the expression of the protein (Tanon Science and Technology Co., Ltd., China).

### Statistical Analysis

The Statistical Analysis software Graphpad Prism 8.3 (Graphpad Software, Inc., San Diego, CA, USA) was applied to analyze and prepare the statistical charts. All data were graphed as the mean ± SEM. Group differences in the escape latency during the Morris water maze test were analyzed using the two-way analysis of variance (ANOVA). Other data were analyzed by one-way ANOVA, followed by Tukey's multiple comparison test to analyze the difference between each group. *P* < *0.05* was considered statistically significant.

## Results

### Sal Improved Aβ1-42 and D-gal/AlCl_3_ Induced Cognitive Dysfunction in Mice

#### Open-Field Test

**Figure 2:**
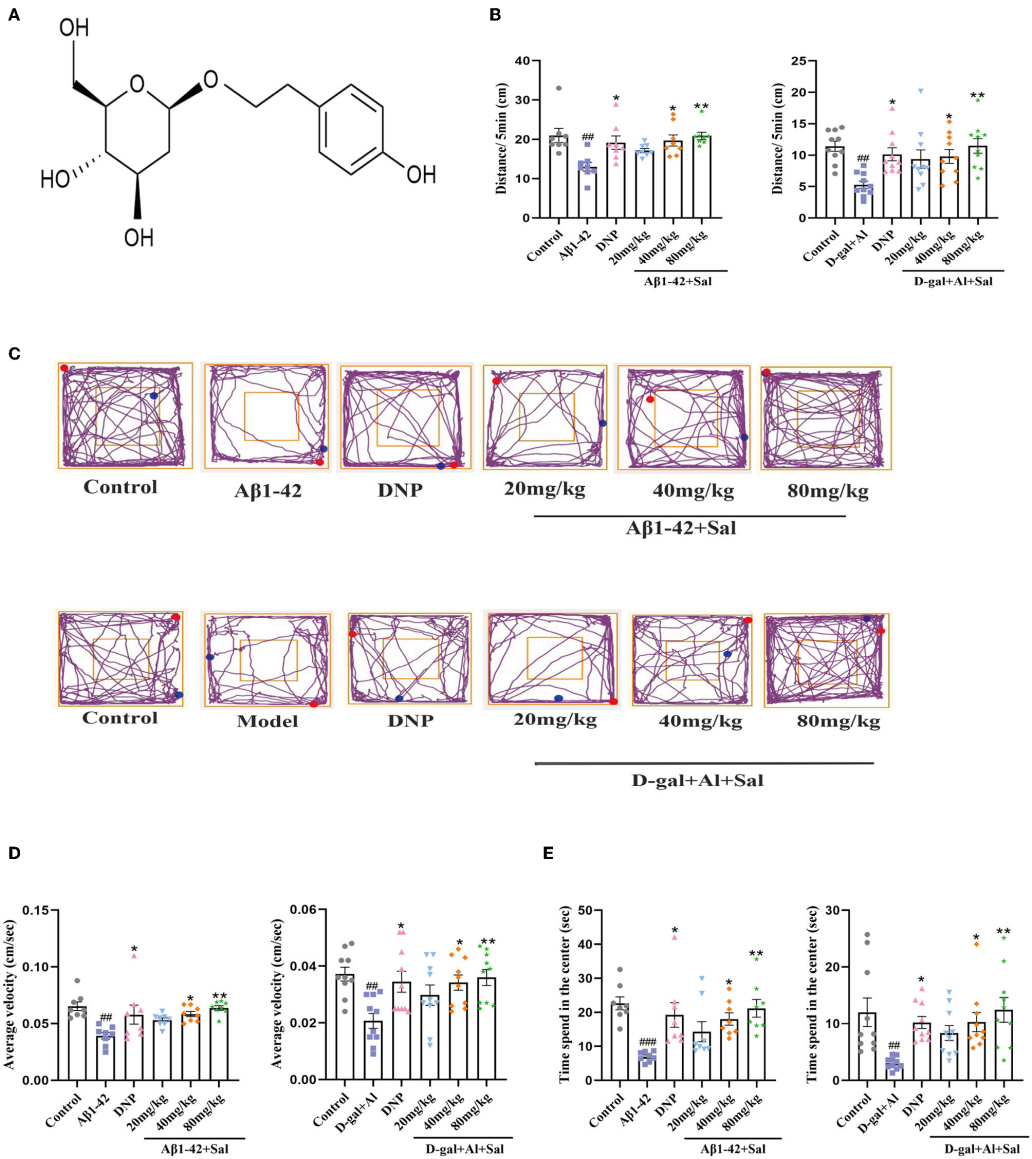
Sal improves the autonomous exploration activities of Aβ1-42 and D-gal/AlCl_3_-induced AD mice. **(A)** The chemical structure diagram of Sal. **(B,D,E)** Mice autonomic capacity test, using open field test. **(B)** Sal or DNP treatment decreased the total distance, **(D)** reduced the average velocity, and **(E)** decreased time spent in the center within 5 min. **(C)** The representative motion track. All data are represented as mean ± SEM (*n* = 8/10). # Significantly different from the control-treated; significantly different from Aβ1-42 and D-gal/AlCl_3_ treated. Significance = ^#^*p* < 0.05, ^##^*p* < 0.01 and ^###^*p* < 0.001, ^*^P < 0.05, ^**^P < 0.01.

An open-field test was used to observe the spontaneous exploration activities of mice. As shown in Figure 2, compared with the control group, there were significant differences in the Aβ1-42 group in the total distance (Figure 2B), the average velocity (Figure 2D), and the intermediate region motion time (Figure 2E), indicating that spontaneous exploration activities defect of Aβ1-42-mice group. Above all, the AD mice treatment by Sal or DNP significantly prolonged the intermediate region motion time and total distance, increasing the average velocity compared to Aβ1-42-mice. The moving track of the mice in the center and surrounding areas is exhibited in Figure 2C. Thus, the results suggested that Sal can substantially improve the autonomous exploration activities of AD mice.

The athletic capacity and anxiety levels of the mice were assessed by OFT (Liu et al., 2021). In the OFT, the AD mice induced by D-gal/AlCl_3_ had fewer total distances than the control mice. The Sal or DNP group exhibited a total distance traveled than the D-gal/AlCl_3_ group (Figure 2B). In terms of the time spent in intermediate quadrant results, the D-gal/AlCl_3_-mice spent less time in intermediate quadrant than the normal mice (Figure 2E). However, the time spent in the middle quadrant was increased in the Sal or DNP group, compared with the D-gal/AlCl_3_ group, and the difference had statistical significance. In addition, the average velocity of the D-gal/AlCl_3_-mice was lower than the control group (Figure 2D). Conversely, the average speed has significantly increased in the Sal or DNP group, compared to the D-gal/AlCl_3_ group. Therefore, the result suggested that Sal can improve athletic capacity and anxiety in AD mice.

#### Morris Water Maze

**Figure 3:**
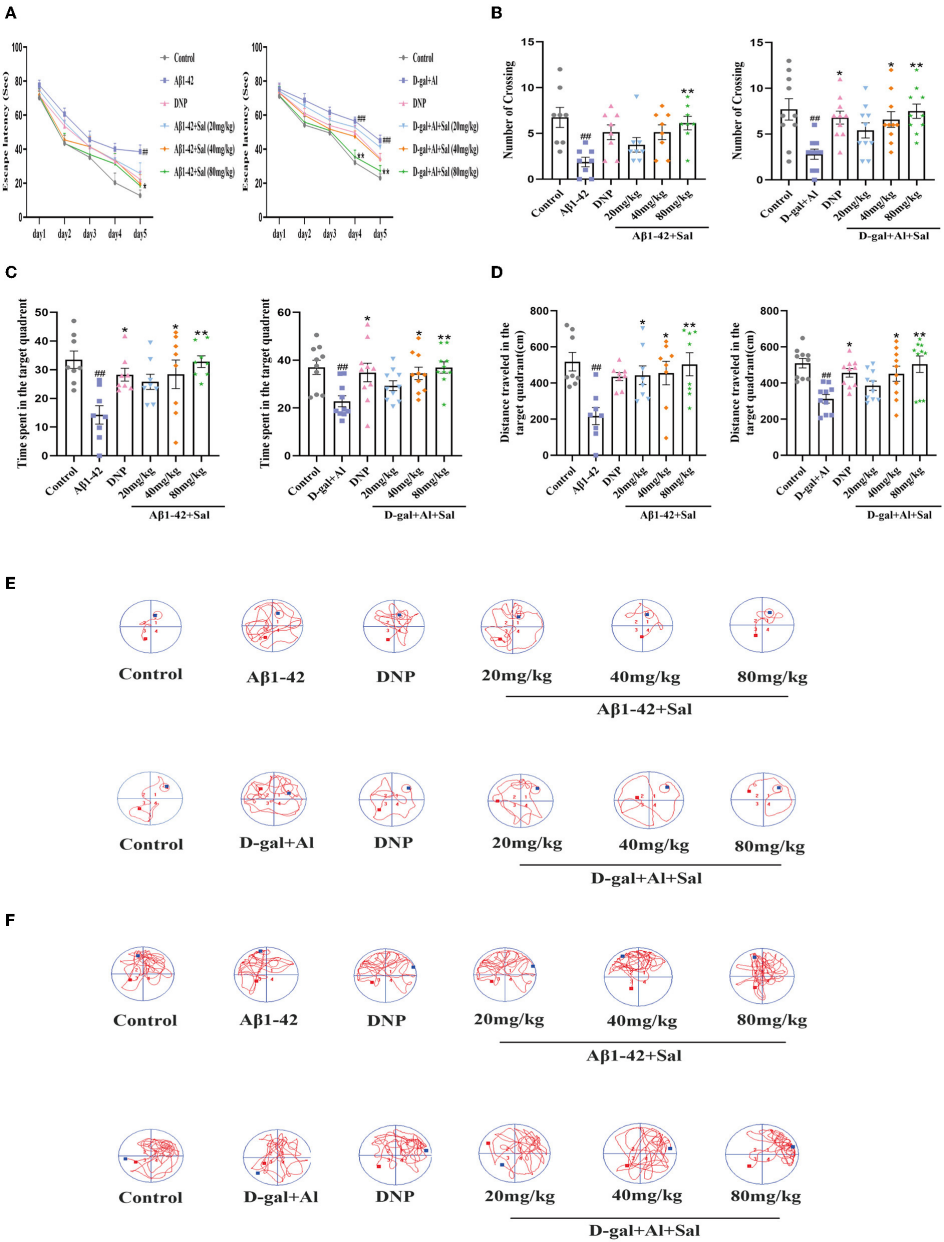
Sal improves learning and memory function in Aβ1-42 and D-gal/AlCl_3_-induced AD mice. The place navigation trial during the MWM. The Sal or DNP-treated AD mice showed a shorter escape latency over 5 days **(A)**. **(B–F)** The spatial probe trial during the MWM. The Sal or DNP-treated AD mice obviously decreased the number of crossings over the platforms **(B)**, reduced the time spent in the target quadrant where the platform was located during the hidden platform training session **(C)**, decreased the distance traveled in the target quadrant **(D)**, improved both the learning curves during the place navigation trial **(E)** and the trajectory of learning on the sixth day **(F)**. Data represent Mean ± SEM and significant differences were considered when ^#^*p* < 0.05, ^##^*p* < 0.01 and ^###^*p* < 0.001 control vs. Aβ1-42 or D-gal/AlCl_3_-induced mice. ^*^P < 0.05, ^**^P < 0.01 vs. Aβ1-42 and D-gal/AlCl_3_ group (*n* = 8/10).

We assessed the learning and memory functions of the AD mice by MWM. First, two-way ANOVA was conducted to analyze the escape latency in the place navigation trial. The results showed that mice treated with Aβ1-42 exhibited prolonged escape latency compared to controls. In contrast, Aβ1-42-mice with Sal or DNP treatment show shorter escape latency than Aβ1-42-mice (Figure 3A). Second, during the spatial probe trial, the one-way ANOVA result indicated that performance was generally worse for the Aβ1-42 group compared with the control group, including a decrease of the number of platform crossings (Figure 3B), curtailing the time spent in the target quadrant (Figure 3C), and reduced the total distance traveled in the target quadrant (Figure 3D). Meanwhile, in this test, Sal and DNP groups where Aβ1-42 was co-administered with Sal or DNP were significantly better than the Aβ1-42 group (Figures 3B–D). Thus, the results revealed that Sal could ameliorate the learning and memory impairments induced by Aβ1-42 in AD mice. The moving track of Aβ1-42 induced mice during the place navigation trial (Figure 3E) and the spatial probe trial (Figure 3F).

To investigate the effect of Sal on the learning and memory function of AD mice induced by D-gal/AlCl_3_, we tested the mice in a Morris Water Maze. This result is consistent with previous reports (Zhang et al., 2019). An increase in escape latency in AD model mice was observed (Figure 3A). In contrast, the escape latency progressively shortened in the Sal or DNP group as time went on. On the sixth day, the number of platform crossings, the time spent in the target quadrant, total distance traveled in the D-gal/AlCl_3_-induced AD mice decreased in the D-gal/AlCl_3_-mice when compared with the control group. Furthermore, the above-mentioned behavior improved after treatment with Sal or DNP (Figures 3B–D). These data suggest that Sal can ameliorate the learning and memory function of D-gal/AlCl_3_-induced AD mice. The moving track of D-gal/AlCl3 induced mice during the place navigation trial (Figure 3E) and the spatial probe trial (Figure 3F).

### Sal Mitigated Aβ1-42 and D-gal/AlCl_3_ Induced Hippocampus Neuronal Injury in Mice

**Figure 4:**
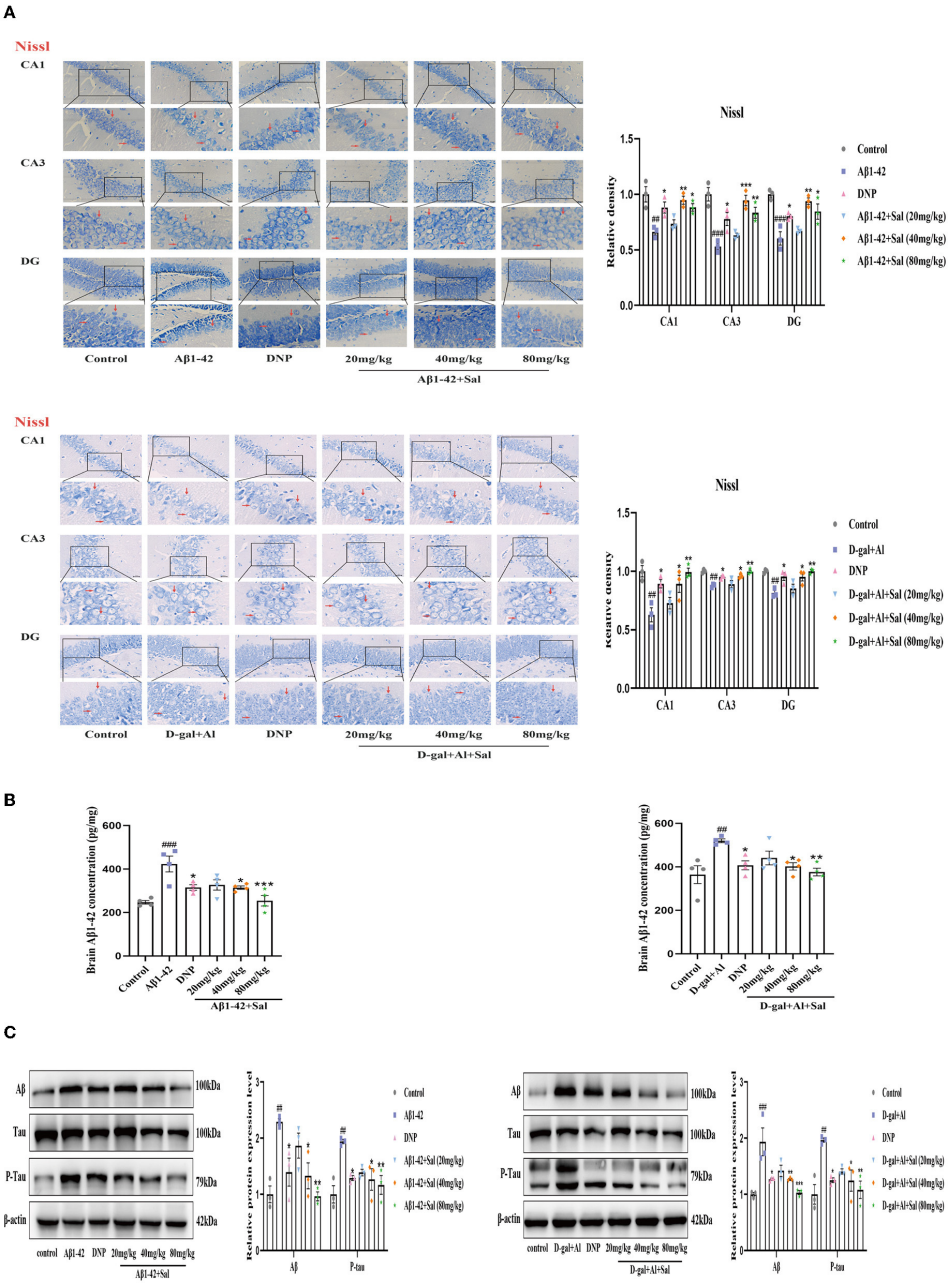
Sal alleviated neuronal injury and downregulated Aβ, P-Tau in Aβ1-42 and D-gal/AlCl_3_-induced AD mice. **(A)** Representative photomicrograph of Nissl staining in the CA1, CA3, and DG regions of the hippocampus of the Aβ1-42 and D-gal/AlCl_3_-treated group. Neuronal damage increased after administration of Aβ1-42 and D-gal/AlCl_3_. Sal or DNP treatment significantly decreased neuronal injury. Nissl staining results are shown by arrows. Scale bar = 25 μm, the magnification of merge is 400 × (*n* = 3). **(B)** Aβ1–42 enzyme-linked immunosorbent assay kits were performed to measure the expression of Aβ1-42 in the hippocampus of AD mice (*n* = 4). **(C)** Brain AD biomarkers (Aβ1-42, Tau, and P-Tau) were determined by Western blotting. Aβ1-42 values were normalized using β-actin, and P-Tau values were normalized using Tau. Data are reported as Mean ± SEM (*n* = 3). ^##^*P* < 0.01, ^###^*P* < 0.001 vs. control group, ^*^P < 0.05, ^**^P < 0.01, ^***^P < 0.001 vs. Aβ1-42 and D-gal/AlCl_3_ group.

The neuron is believed to play a vital role in learning and memory functions. We, therefore, analyzed the number of neuronal in the CA1, CA3, and DG regions of the hippocampus by Nissl staining to investigate the effects of Sal treatment on the neuronal morphology change. The neurons in the hippocampus area were in a larger quantity, arranged in neat rows, and rich in Nissl Bodies in the control group. In contrast, the neurons were decreased in quantity, arranged in a disorderedly fashion, with an obscure or disappeared karyopyknosis, or karyolysis, and poor in Nissl Bodies in the Aβ1-42 group (Figure 4A). The Sal or DNP-treated groups reversed the above pathological features, namely, the neurons were high in quantity, arranged neatly, with more Nissl Bodies, little karyopyknosis, and karyolysis as seen in Figure 4A. All in all, these data demonstrated that Sal could attenuate its neuronal death.

To determine whether Sal protects the neurons from D-gal/AlCl_3_-induced damage, we performed Nissl staining to analyze the shape of neurons in the hippocampus of AD mice. Nissl staining revealed that D-gal/AlCl_3_-mice exhibited neuronal pyknosis and significantly reduced neuronal counts. Conversely, in the Sal or DNP group, the above mentioned pathological features were ameliorated, and the density of neurons also increased significantly (Figure 4A). Together, these results indicate that Sal ameliorated the damage of neurons induced by D-gal/AlCl_3_.

### Sal Decreased Aβ1-42 and D-gal/AlCl_3_ Induced the Increased of Aβ1-42 and P-Tau in Mice

**Figure 5:**
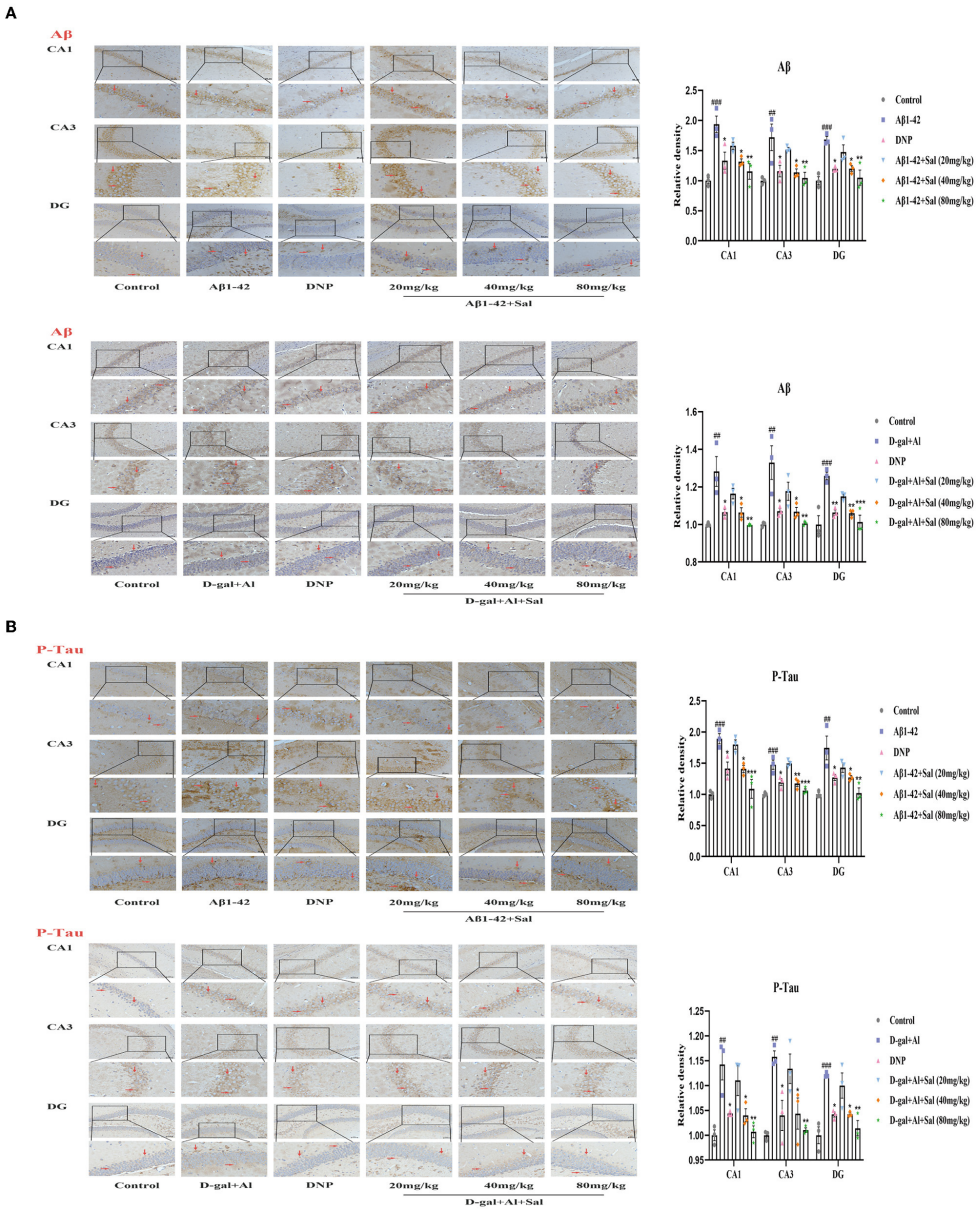
Sal reduced Aβ, P-Tau in Aβ1-42 and D-gal/AlCl_3_-induced AD mice. Immunochemical staining of Aβ **(A)** and P-Tau **(B)** in the CA1, CA3, and DG regions of the hippocampus, representative of the relative density of related protein in the hippocampus. Aβ and P-Tau staining results are shown by arrows. Scale bar = 50 μm, the magnification of merge is 200 ×. All data are represented as mean ± SEM (*n* = 3). ^##^*P* < 0.01, ^###^*P* < 0.001 vs. control group, ^*^P < 0.05, ^**^P < 0.01, ^***^P < 0.001 vs. Aβ1-42 and D-gal/AlCl_3_ group.

Aggregation of both Aβ and hyperphosphorylated Tau protein is a primary hallmark of AD (Bakota and Brandt, 2016). To explore the effect of Sal on Aβ1-42-mice, we examined the expression of Aβ and p-Tau in the brain of AD mice by Enzyme-linked immunoassay kits (ELISA), Immunohistochemical staining, and Western blotting analysis. Figures 4, 5 exhibit that the level of Aβ was significantly increased for the Aβ1-42-mice compared with the regular group, as measured by the ELISA kit. This data indicates that the AD model was prepared successfully. In addition, the levels of Aβ1-42 in the Sal or DNP group were lower compared with Aβ1-42-mice (Figure 4B), suggesting that Sal treatment was not inferior to DNP. The expression of Aβ and P-Tau protein was upregulated in the Aβ1-42 group, as indicated by western blotting while the presentation of Aβ1-42, p-Tau dropped in the DNP group. For comparison, this downregulate tendency was more pronounced in the Sal group (Figure 4C). Furthermore, Immunohistochemical staining showed that Aβ and p-Tau positive areas were significantly increased in the hippocampal region of the Aβ1-42 group, with a significant difference compared with the regular group. Increased yellowish-brown Aβ plaque deposition and brown P-Tau deposits in the model group. Conversely, treatment with Sal or DNP marked reduced the positive area of Aβ and P-Tau in the hippocampal region, the yellowish-brown Aβ plaque deposition and brown P-Tau deposits are both decreased, with a significant difference compared with the Aβ1-42 group (Figures 5A,B). In short, the above results demonstrated that Sal could exert therapeutic effects on AD by directly reducing both deposition of the Aβ peptide levels and the hyperphosphorylation of Tau.

To determine whether Sal treatment can decrease the Aβ accumulation and hyperphosphorylation of Tau in D-gal/AlCl_3_-mice, we first carried out analyses by ELISA kits. The results indicated that the deposition of Aβ increased in the model group, which implied that the AD model was established successfully. Nevertheless, Sal, DNP groups all presented reductions in the Aβ deposition compared with the D-gal/AlCl_3_ group, meaning Sal has similar efficacy to DNP to exert therapy effect on AD (Figure 4B). Next, to further confirm the impact of Sal on the accumulation of Aβ and hyperphosphorylation of Tau in AD mice, the relative expression levels of Tau, p-Tau, and Aβ in the brain of AD mice were examined by Western blotting (Figure 4C). The result demonstrated that the expressions of p-Tau and Aβ were reduced in the Sal or DNP group, and the statistical significance of the differences for comparison D-gal/AlCl_3_ group. Finally, IHC was performed. As shown in Figures 5A,B, the yellowish-brown Aβ plaque deposition and brown P-Tau deposits are both decreased in the Sal or DNP group. In summary, these findings suggest that Sal can improve the Aβ loaded and phosphorylation of Tau and subsequent pathology of D-gal/AlCl_3_-mice.

### Sal Inhibited Aβ1-42 and D-gal/AlCl_3_ Induced Pyroptosis in Mice

**Figure 6:**
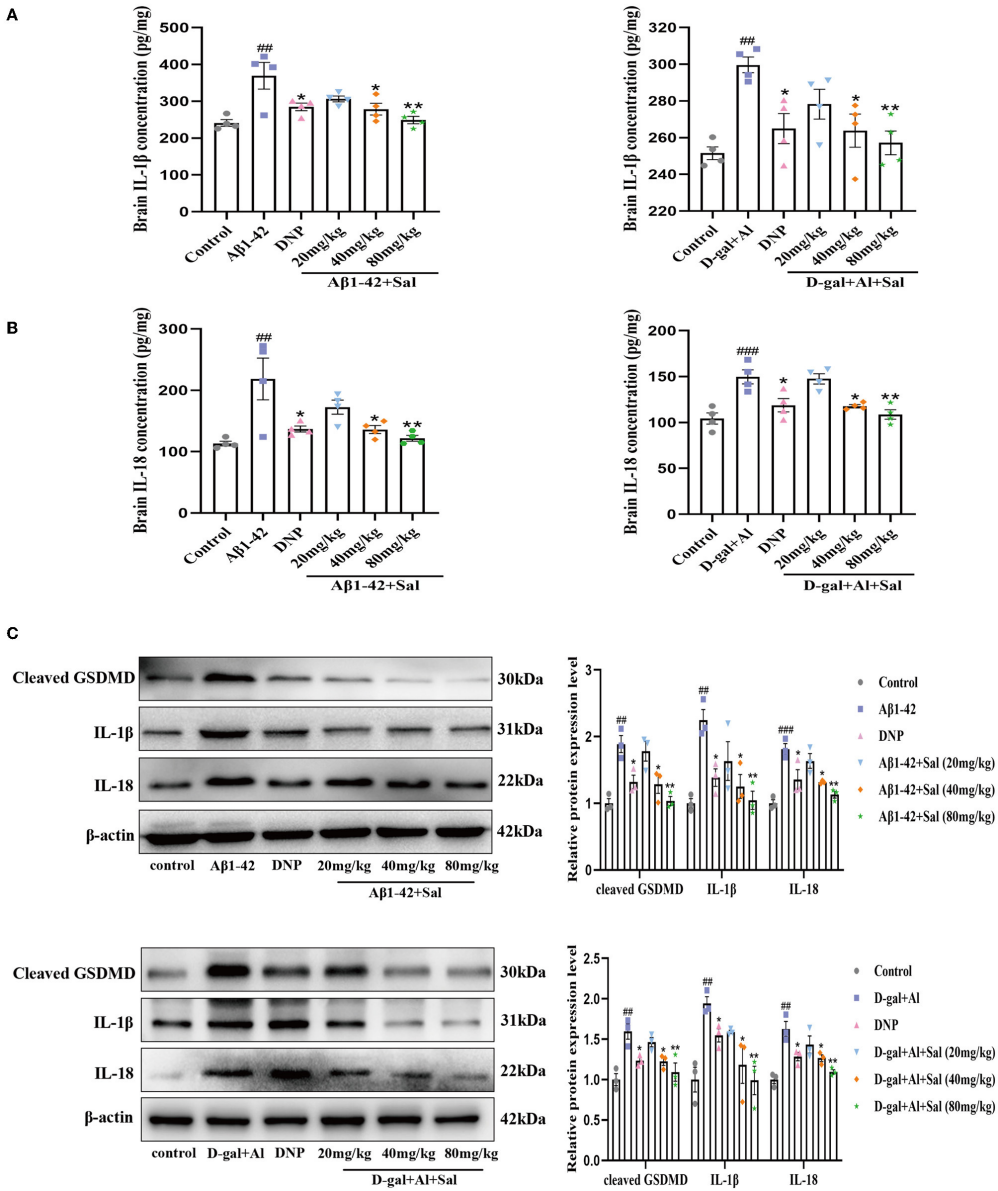
Sal mitigated pyroptosis in Aβ1-42 and D-gal/AlCl_3_-induced AD mice. Aβ1-42 and D-gal/AlCl_3_-induced AD mice brain expression of IL-1β **(A)** and IL-18 **(B)** analyzed by enzyme-linked immunosorbent assay (ELISA) kits (*n* = 4). **(C)** The Western blot analysis of IL-1β, IL-18, and cleaved GSDMD in the hippocampus of the AD mice (*n* = 3). Image J was applied to quantify the bands, and a histogram represents the differences after normalization to β-actin. All data are represented as mean ± SEM. ^##^*P* < 0.01, ^###^*P* < 0.001 vs. control group, ^*^P < 0.05, ^**^P < 0.01, vs. Aβ1-42 and D-gal/AlCl_3_ group.

In the present study, ELISA was used to evaluate the inflammatory cytokines, namely, IL-1β and IL-18. Based on the ELISA results, the expression level of IL-1β and IL-18 were significantly elevated in the brain of AD mice induced by Aβ1-42, suggesting that IL-1β and IL-18 are cytokines that play crucial roles in pyroptosis and the onset of AD. As Figure 6 shows, the Aβ1-42-mice treat applied Sal, the expression of IL-1β and IL-18 was significantly lower than that in the Aβ1-42 group (Figures 6A,B). Subsequently, we further investigated the expression of pyroptosis-related proteins, that is to say, IL-1β, IL-18, and cleaved GSDMD by western blotting. Results demonstrated that the protein level of IL-1β, IL-18, and cleaved GSDMD increased in the Aβ1-42 group compared with the regular group (Figure 6C). In comparison, the Sal group found a markedly reduced expression level of IL-1β, IL-18, and cleaved GSDMD. Notably, GSDMD-N was cleaved by activated GSDMD, which could motivate the secretion of IL-1β, IL-18, and subsequently drive pyroptosis (He et al., 2015). These results imply that pyroptosis is closely related to AD, and Sal could prevent pyroptosis in Aβ1-42-induced AD mice.

GSDMD is a crucial biomarker of pyroptosis. To explore the action and mechanism of Sal in pyroptosis, we assessed both the IL-1β and IL-18 and the expression of GSDMD proteins related to the pyroptosis by ELISA kits and Western blotting analysis. ELISA results indicated that treatment with Sal gradually downregulated the secretion of IL-1β and IL-18 induced by D-gal/AlCl_3_ (Figures 6A,B). Western blot further confirmed our results. The result showed that the protein levels of IL-1β. IL-18, and cleaved GSDMD decreased in the Sal group (Figure 6C). These results demonstrate that pyroptosis may involve AD onset, and Sal could ameliorate the course of AD through inhibited pyroptosis.

### Sal Attenuated Aβ1-42-Induced Pyroptosis Through Modulation of the NLRP3/Caspase-1 Signaling Pathways in Mice

**Figure 7:**
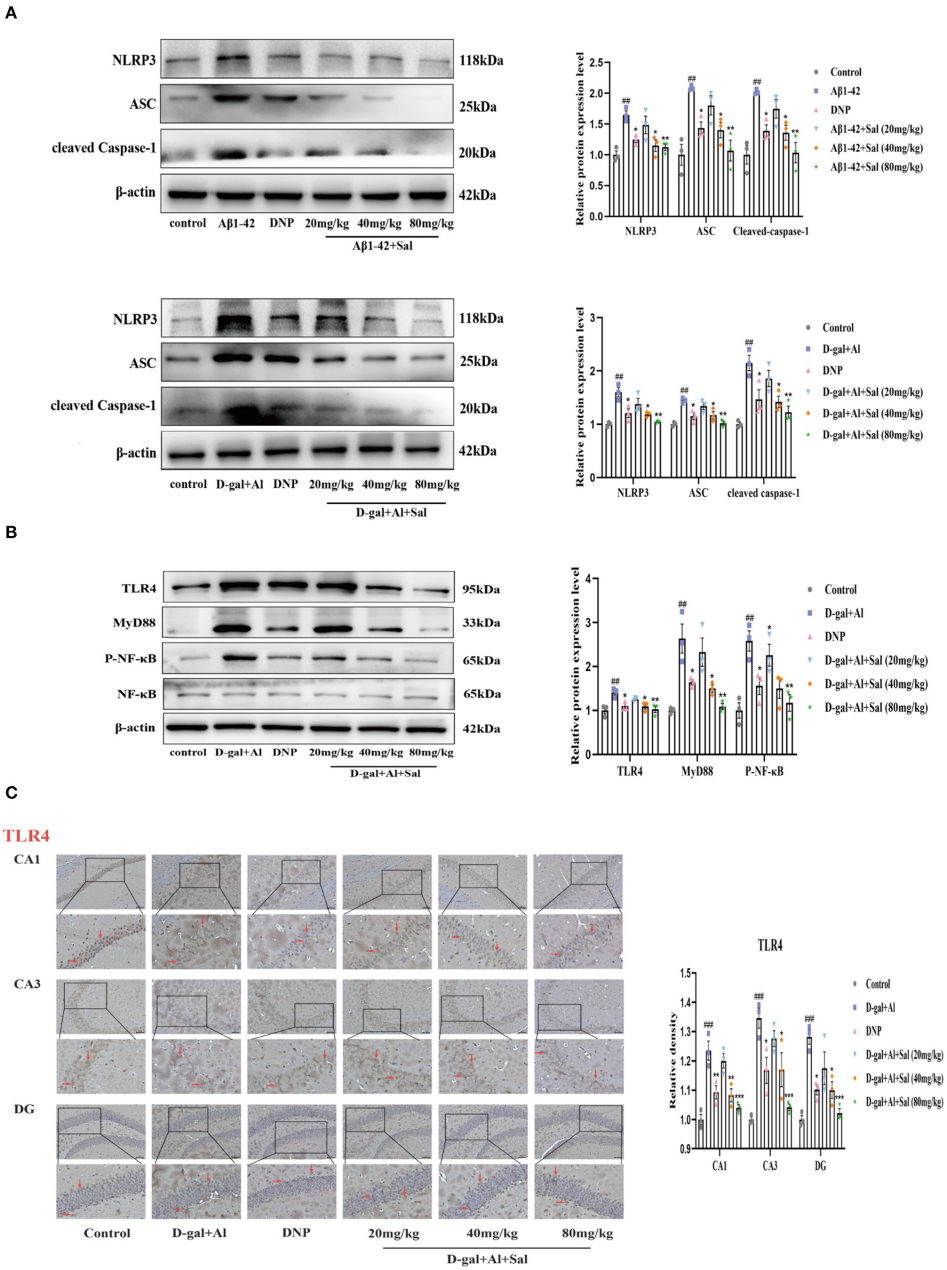
Sal suppressed pyroptosis in Aβ1-42 and D-gal/AlCl_3_-induced AD mice via NLRP3/Caspase-1 or TLR4/NF-κB/NLRP3/Caspase-1 signaling pathway. **(A,B)** Western blot analysis of TLR4, MyD88, p-NF-κB, NF-κB, NLRP3, ASC, and cleaved caspase-1. p-NF-κB was normalized with NF-κB and others were normalized with β-actin. **(C)** Immunochemical staining of TLR4 in the CA1, CA3, and DG regions of the hippocampus of AD mice brains, and the IHC staining results are shown by arrows; scale bar = 50 μm, Original magnification: x200. Quantitative analyses of the TLR4 positive area in the hippocampus by Image J. Values are expressed as mean ± SEM (*n* = 3). ^##^*P* < 0.01, ^###^*P* < 0.001, vs. control group, ^*^P < 0.05, ^**^P < 0.01, ^***^P < 0.01 vs. Aβ1-42 and D-gal/AlCl_3_ group.

**Figure 8:**
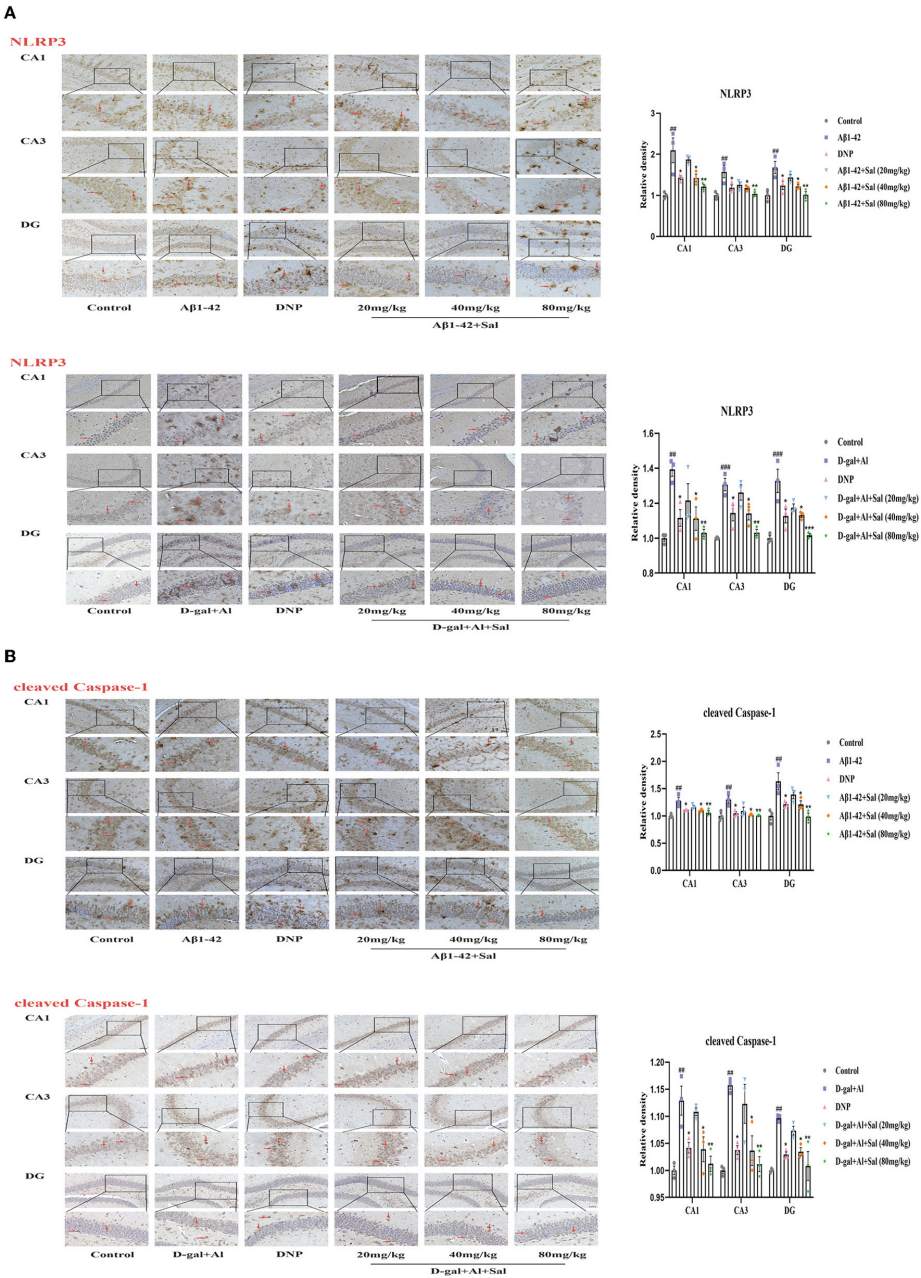
Sal suppressed pyroptosis in Aβ1-42 and D-gal/AlCl_3_-induced AD mice via NLRP3/Caspase-1 or TLR4/NF-κB/NLRP3/Caspase-1 signaling pathway. Immunochemical staining of NLRP3 **(A)**, cleaved caspase-1 **(B)** in the CA1, CA3, and DG regions of the hippocampus of AD mice brains, and the IHC staining results are shown by arrows; scale bar = 50 μm, Original magnification: x200. Quantitative analyses of the NLRP3, cleaved caspase-1 positive area in the hippocampus by Image J. Values are expressed as mean ± SEM (*n* = 3). ^##^*P* < 0.01, ^###^*P* < 0.001, vs. control group, ^*^P < 0.05, ^**^P < 0.01, ^***^P < 0.01 vs. Aβ1-42 and D-gal/AlCl_3_ group.

To explore Sal's action and underlying mechanism in pyroptosis induced by Aβ1-42, we validated it using IHC and western blotting. The western blotting results suggested that the expression of the proteins of NLRP3, ASC and cleaved Caspase-1 levels were increased in the Aβ1-42-group (Figure 7A), the high expression of these proteins could be inhibited by administration with Sal. Consistently, the IHC exhibited that the increased brown NLRP3, cleaved Caspase-1 deposits were seen in the hippocampus of AD mice, and the brown deposits were suppressed with Sal, which indicates Sal can inhibit NLRP3/Caspase-1 (Figures 8A,B). Thus, the above data show that Sal can mitigate pyroptosis by inhibiting the NLRP3/caspase-1 signaling pathway.

### Sal Attenuated D-gal/AlCl_3_-Induced Pyroptosis Through Modulation of the TLR4/NF-κB/MyD88/NLRP3 and NLRP3/Caspase-1 Signaling Pathways in Mice

Aβ1-42 models for the potential mechanisms of Sal have revealed that Sal can ameliorate the pathological features of AD via inhibited pyroptosis. To further confirm how Sal modulates pyroptosis, and in turn, intervenes in the pathogenesis of AD, we validated the results obtained from the Aβ1-42 mice model using the D-gal/AlCl_3_ mice model. As expected, Western blotting results showed an increase of TLR4, MyD88, p-NF-κB, NLRP3, ASC, and Cleaved Caspase-1 protein expression levels in the model group. Conversely, these protein expressions were reduced in the Sal group (Figures 7A,B). Furthermore, the result of IHC is consistent with Aβ1-42-mice (Figures 7C, 8A,B). Normal brain tissue sections showed no significant positive expression under high magnification and increased brown TLR4, NLRP3, cleaved Caspase-1 deposits were seen in the hippocampus of AD mice, and small amounts of brown deposits were seen in the Sal groups. Thus, the above experiments verified that Sal inhibits pyroptosis by suppressing the TLR4/MyD88/NF-κB/NLRP3 and NLRP3/Caspase-1 signaling pathway.

### Sal Protected PC12 Cells Against D-gal-Induced Cytotoxicity and Improved Viability

**Figure 9:**
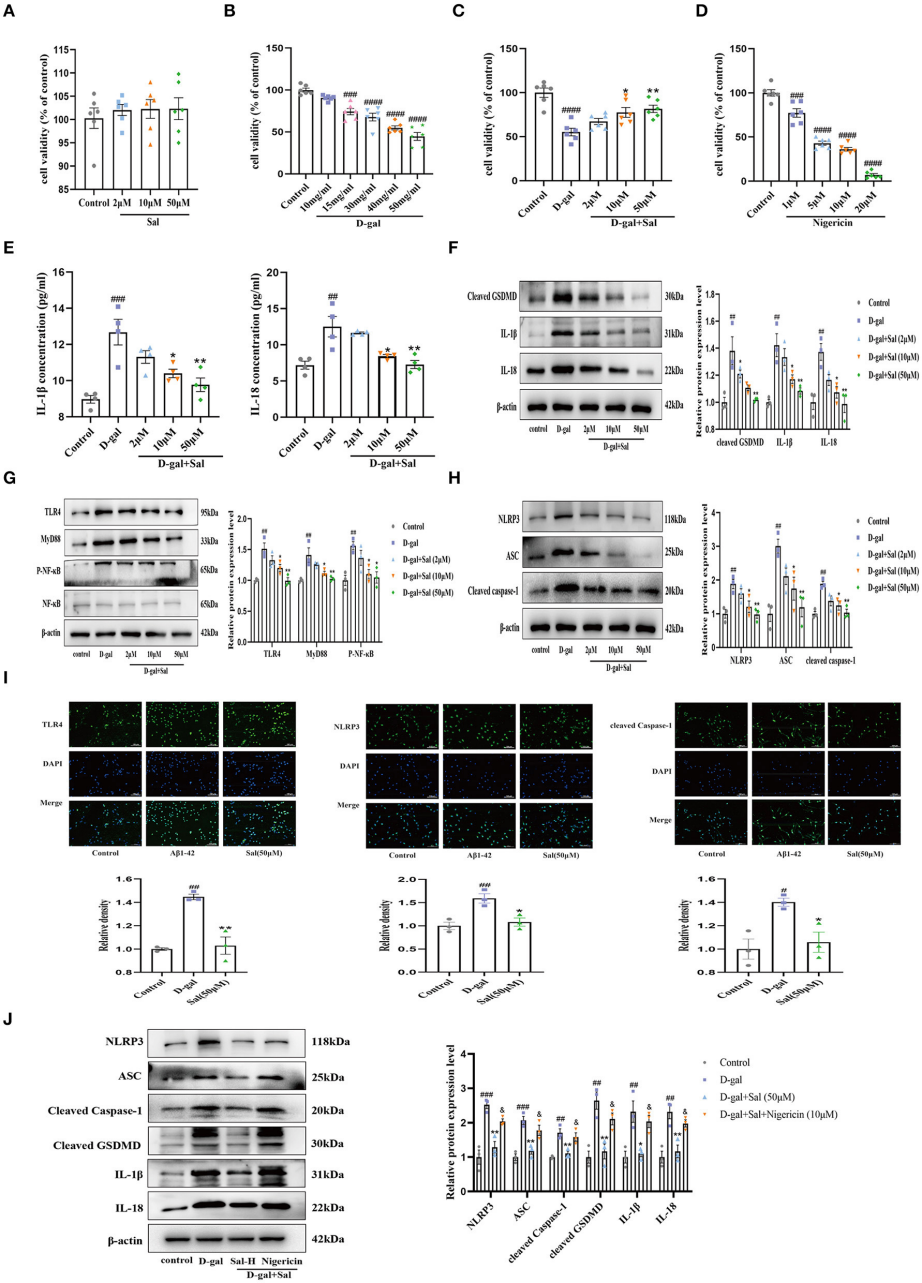
Sal regulated PC-12 cell pyroptosis via inhibiting the TLR4/NF-κB/NLRP3/ASC/Caspase-1 signaling pathways. **(A)** CCK-8 measured the cytotoxic effects of Sal on PC12 cells; Sal treatment has no significant cytotoxicity on PC12 cells (*n* = 6). **(B,C,E)** CCK-8 conducted the assays on PC12 cells viability after the cells were pre-treated with Sal for 2 h, and then added D-gal for 48 h. **(B)** PC12 cells Viability was decreased as the increased concentration of D-gal (*n* = 6). **(C)** Sal treatment dose-dependently prevented PC12 cells from damage (*n* = 6). **(D)** With the increase of Nigericin concentration, the viability of PC12 cells decreased gradually (*n* = 6). **(E)** IL-1β and IL-18 levels in the cell supernatant by ELISA (*n* = 4). **(F)** The Western blot analysis of cleaved GSDMD, IL-1β, and IL-18 of PC12 cells (*n* = 3). **(G)** Immunoblots of TLR4, MyD88, NF-κB and, p-NF-κB. p-NF-κB was normalized with NF-κB and others were normalized with β-actin. **(H)** The expression of NLRP3, ASC, and cleaved Caspase-1 of PC12 cells was detected by western blotting (*n* = 3). **(I)** PC12 cells were pre-treated with Sal for 2 h, following added D-gal for 6 h. Then immunofluorescence staining using specific antibody TLR4, NLRP3, and cleaved Caspase-1 (green) immunofluorescence. Scale bar, 50 μm, the magnification of merge is 200 × (*n* = 3). **(J)** Western blot was used to examine the expressions of NLRP3, ASC, cleaved Caspase-1, cleaved GSDMD, IL-1β, and IL-18 in the different groups (*n* = 3). Image J was used to quantify the bands, and a histogram represents the differences after normalization to β-actin. All the results are shown as means ± SEM. ^#^*P* < 0.05, ^##^*P* < 0.01, ^###^*P* < 0.001, ^####^*P* < 0.0001 vs. control group, ^*^P < 0.05, ^**^P < 0.01 vs. D-gal group. ^&^*P* < 0.05 vs. Sal-H group.

To screen an optimum action concentration of D-gal, we first added D-gal (10, 15, 30, 40, 50 mg/ml) to 96-well culture plates and allowed it to act on the PC12 cells for 48 h. As Figure 9 shows, cell viability decreased with the increase of D-gal action concentration, detected by the CCK8 assay (Figure 9B). Finally, D-gal (15 mg/ml), was selected as working concentrations for the subsequent experiments.

To explore the action of Sal in D-gal induced cytotoxic and neuroprotective effects on PC12 cells, assays were performed using a CCK-8 assay. As shown in Figure 9, after PC12 cells were exposed to Sal (2, 10, 50 μM) for 24 h, there were no significant hits for the viability of PC-12 cells (Figure 9A), suggesting that Sal did not alter the survival of PC12 cells. Subsequently, 2, 10, and 50 μM of Sal were added to the culture media, and cells were pre-incubated for 2 h. Then, the cells were incubated with D-gal (15 mg/ml) for 48 h, finally using CCK-8 for analyses. As shown in Figure 9C, the percentage of PC12 cells was significantly increased in the Sal-treated group compared to the D-gal group. The result implied that Sal might act to prevent PC12 cells from damage.

### Protective Effects of Sal Against PC12 Cells Damage Induced by D-gal *via* Regulating TLR4/NF-κB/NLRP3/Caspase-1 Signaling Pathway

To explore the underlying mechanisms of D-gal induced PC12 cells damage, ELISA was applied to determine the secretion of IL-1β and IL-18. Results indicated that Sal considerably decreased the expression of IL-18 and IL-1β in D-gal-induced PC12 cells, suggesting that Sal can exert mitigated pyroptosis via the decreased release of inflammatory factors (Figure 9E). Meanwhile, Western blotting analysis was used to analyze the TLR4-related and the NLRP3-related signaling pathway protein to verify the above hypothesis further. Consistent results were obtained. Protein expression was downregulated in the Sal group, including TLR4, MyD88, p-NF-κB, NLRP3, ASC, Cleaved Caspase-1, Cleaved GSDMD, IL-1β, and IL-18 (Figures 9F–H). Furthermore, immunofluorescence assays confirmed that the expression of TLR4, NLRP3, and cleaved Caspase-1 were significantly reduced by treatment with Sal (Figure 9I). The above result is consistent with the previous two AD mice model results, indicating that Sal could suppress pyroptosis by inhibiting TLR4/MyD88/NF-κB/NLRP3 and NLRP3/Caspase-1 signaling pathways in D-gal-induced PC-12 cells.

### Effect of Sal on Nigericin-Induced NLRP3 Activation in PC12 Cells

CCK-8 assays were conducted to screen a suitable concentration of Nigericin for our experiment. The Nigericin concentration chosen for this screening was 10 μM (Figure 9D).

As demonstrated for Aβ1-42 and D-gal/AlCl_3_ AD mice models and PC12 cells model, pyroptosis plays a critical role in the progression of various nervous system diseases (such as AD, PD, HD, and ALS) (Voet et al., 2019). In the present experiment, we aimed to explore the mechanism of Sal during the pathogenesis of AD. To further explore the action of Sal on NLRP3 activation, Western blotting was used to assess changes in NLRP3 and its downstream proteins in Nigericin + Sal-treated PC12 cells. As indicated in the figure, the level of NLRP3 and its downstream proteins was noticeably increased in the D-gal group compared to the normal group. By contrast, the level of NLRP3 was decreased in the Sal group compared with the D-gal group. Interestingly, compared with the Sal group, the Nigericin group efficiently reversed the reduced level of NLRP3 effectively while increasing the level of ASC, cleaved Caspase-1, cleaved GSDMD, IL-1β, and IL-18, a downstream target of NLRP3 (Figure 9J). Thus, the above results confirmed that Sal inhibited pyroptosis mainly through targeting suppression of the NLRP3 inflammasome.

### NLRP3 is Involved in the Inhibitory Effect of Sal on D-gal-Induced PC12 Cells Pyroptosis

**Figure 10:**
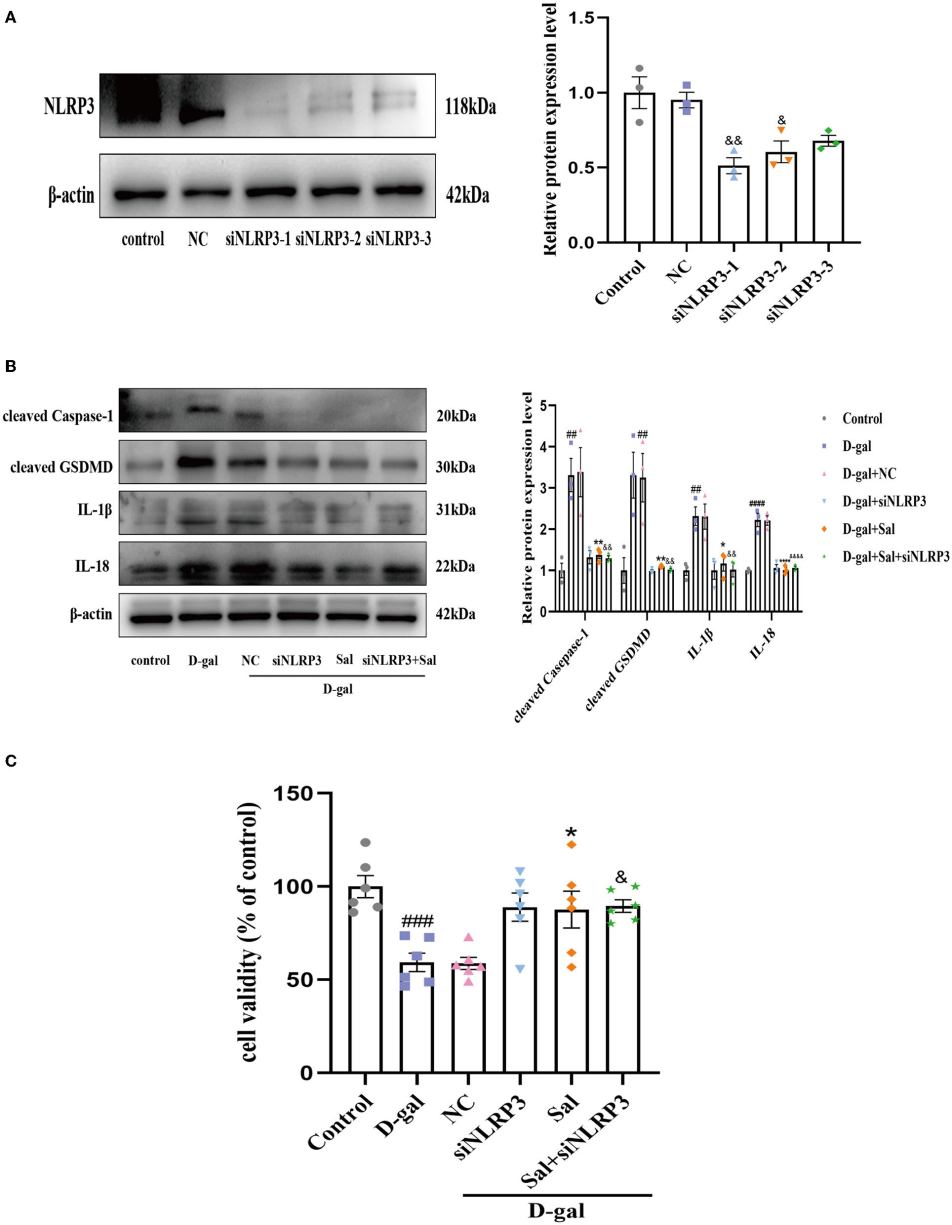
Sal inhibited pyroptosis through NLRP3 inflamasome. **(A)** Western blot validated the effect of NLRP3 knockout, β-actin was used as an internal control (*n* = 3). **(B)** The Western blot analysis of cleaved Caspase-1, cleaved GSDMD, IL-1β, and IL-18 of PC12 cells, β-actin was used as an internal control (*n* = 3). **(C)** CCK-8 measured the cytotoxic effects of Sal or siNLRP3 on PC12 cells (*n* = 6). ^##^*P* < 0.01, ^###^*P* < 0.001, ^####^*P* < 0.0001 vs. control group, ^*^P < 0.05, ^**^P < 0.01 vs. D-gal group. ^&^*P* < 0.05, ^&&^*P* < 0.01, ^&&&&^*P* < 0.0001 *vs*. NC group.

We hypothesized that Sal acts against D-gal-induced NLRP3 inflammasome activation by downregulating NLRP3. To test this, we knocked down NLRP3 expression in PC12 cells by siRNA. Firstly, the Western blot result showed that the sequence 1 of NLRP3 siRNA had a better effect in silencing NLRP3 (Figure 10A). As shown in Figure 10B, we found that the NLRP3 siRNA group or Salidroside significantly reversed D-gal induces upregulation of cleaved Caspase-1, GSDMD, IL-1β, and IL-18, further suggesting that NLRP3 inflammasome-mediated pyroptosis plays a central role in AD. At the same time, compared with the NLRP3 siRNA group, the Salidroside combined NLRP3 siRNA group failed to change the protein expression of cleaved Caspase-1, GSDMD, IL-1β, and IL-18 in PC12 cells (Figure 10B). Moreover, NLRP3 siRNA or Salidroside rescued in cell viability induced by D-gal, and the Salidroside combined NLRP3 siRNA group failed to change cell viability compared with NLRP3 siRNA (Figure 10C). Collectively, the above data demonstrate that Salidroside inhibited pyroptosis by targeting the NLRP3 inflammasome.

## Discussion

AD is the most common cause of dementia and the fifth-largest cause of death globally (Cortes-Canteli and Iadecola, 2020). Presently, the most significant number of AD patients in the world are in China. It is therefore vital that the potential pathogenesis of AD is elucidated and potential therapeutic drugs are developed. Previous studies have elucidated that the pathogenesis of AD may be related to abnormal Aβ deposition, Tau hyperphosphorylation, cholinergic hypothesis, and inflammatory response (Sharma et al., 2019). Nonetheless, the precise mechanism of AD remains ambiguous. Recent studies have proposed that pyroptosis can trigger AD, leading to brain lesions and further lured synaptic dysfunction, neuronal degeneration, and memory impairment (El-Sisi et al., 2021). The present study was designed to verify whether: (1) the NLRP3 inflammasome-mediated pyroptosis contributes to the pathogenesis of AD; and (2) to examine if Sal can prevent neurons from damage in Aβ1-42 and D-gal/AlCl_3_-induced AD mouse models and D-gal-treated PC12 cell models through inhibited NLRP3 inflammasome-mediated pyroptosis.

To gain insight into AD etiopathogenesis, we established AD models *in vivo* and *in vitro* to find the link between AD and pyroptosis. Related researches have shown that intracerebroventricular (icv) injections of Aβ1-42 peptide can mimic some of the symptoms of AD, including memory impairment, oxidative stress, and neuroinflammation (Fu et al., 2019). Based on this, we established an AD model by injecting Aβ1-42 into the bilateral hippocampus, as other articles (Chen et al., 2021) have outlined. The results were in line with prior research (Li et al., 2018) findings for AD mice treated by Sal, which exhibited reduced escape latency. The number of crossings and time spent in the target quadrant was increased and significantly reversed Aβ1-42-induced cognitive impairment in AD mice. Consistent with previous studies (Bassil et al., 2020), our study indicates that Aβ and p-Tau positive areas increased in AD mice induced by Aβ1-42. The treatment effects of Sal were consistent with DNP, where both Aβ and p-Tau positive areas were reduced, hinting that Sal can play a therapeutic role in AD pathogenesis. Moreover, the number of neurons in the hippocampus's CA1, CA3, and DG regions were reduced when injected with Aβ1-42, which is caused by accumulated Aβ peptide activating the NLRP3 inflammasome and triggering pyroptosis. As described in a previous study (Dempsey et al., 2017), Aβ accumulates in the AD brain to form characteristic plaques and then activates the NLRP3 inflammasome, triggering the pyroptosis via the NLRP3/caspase-1/GSDMD signaling pathway, and finally leading to levels of inflammatory factors IL-1β and IL-18 becoming elevated. Our result indicates that Sal significantly offset the increase of IL-1β and IL-18 expression induced by Aβ1-42 and inhibited the expression of the pyroptosis-related protein, namely, NLRP3, ASC, cleaved Caspase-1, cleaved GSDMD, IL-1β, and IL-18, its mechanism may act by inhibiting the NLRP3/caspase-1 signaling pathway.

This AD model induced by Aβ1-42 with bilateral hippocampal injection was a short-term model that articulates AD's pathogenesis only from the amyloid hypothesis, meaning it has some limitations. Therefore, to ensure the reliability of the efficacy of Sal, as outlined in previous research (Zhang W. et al., 2020; Liu et al., 2021), D-gal/AlCl_3_ was used to establish a long-term AD mice model. Meanwhile, the D-gal-induced PC12 cell damage model was established, further validating Sal's role in AD and the exact mechanism of action.

D-gal is a drug that can induce immune system defects, oxidative stress damage, and neurochemical changes, and these neurochemical changes are similar to normal aging in animals (Wang J. et al., 2017). The metabolite in the brain of high-level D-gal was Galactose, and its accumulation in neurons may result in a destroyed balance of cell osmotic pressure and changed cell morphology, loss of neurons, and even AD occurs (Wei et al., 2017). Furthermore, it is known that Al is a neurotoxic agent, and long-term exposure to Al can cause severe damage to hippocampal cell structures, leading to the development of neurodegenerative diseases (Abd-Elhady et al., 2013). The AD mice model induced by D-gal/AlCl_3_ replicates the whole aging process and triggers AD-like symptoms, including cognitive and memory defects, excessive amyloid protein expression, oxidative damage, and inflammation (Zhang W. et al., 2020). PC12 was classified as the adrenal pheochromocytoma cell line, widely used to study the pathogenesis and progression of various neurological diseases, such as AD, Parkinson's disease (PD), epilepsy, and ischemia (Thiel et al., 2015; Loeffler et al., 2016; Ivashko-Pachima and Gozes, 2018; Lahiani et al., 2018). Thence, to build a model closer to a human AD model, we established an AD mouse model induced by D-gal/AlCl_3_. Besides, the D-gal-induced PC12 cell injury model was established to further elucidate roles for pyroptosis in AD pathogenesis.

As is well-known to us, D-gal combined with AlCl_3_ can induce Aβ accumulation, Tau hyperphosphorylation, and neuroinflammatory responses (Chiroma et al., 2019), leading to the increased expression of inflammatory factors IL-1β and IL-18. Aβ was intracerebral DAMP that can be recognized by microglia surface PRRs, Toll-like receptors, NOD-like receptors (NLRs), RAGE, and SRs, activated downstream signal transduction pathways produce effects (Salminen et al., 2009). Toll-like receptor 4 (TLR4) was a pattern recognition receptor (PRR) expressed mainly on the surface of microglia (Liu Y. et al., 2020), associated with the pathogenesis of AD, PD, and ischemia-reperfusion diseases. TLR4 activation leads to its downstream recruitment via the upstream adaptor molecule myeloid differentiation primary response 88 (MyD88) to activate NF-κB and increase levels of several pro-inflammatory factors such as IL-1β and TNF-α (Liu M. et al., 2020). Activated NF-κB translocates from the cytoplasm to the nucleus and then promotes the secretion of IL-1β and IL-18, which are crucial to regulating pyroptosis (Liu et al., 2017). Meanwhile, multiple studies have shown that NF-κB is an essential upstream activator of the NLRP3 inflammasome, triggering the initiation and assembly of inflammasome through induction of NLRP3 expression and promoting the inflammatory cascade response (Guo et al., 2015; Cui et al., 2020). The assembly of NLRP3 inflammasome activated caspase-1, which further drives the cleavage of GSDMD, generated N-terminal fragments, and then induced membrane pore formation, promoted the release of IL-1β and IL-18, and finally leads to pyroptosis (Kesavardhana and Kanneganti, 2017). Our results demonstrate that D-gal/AlCl_3_-mice after Sal treatment had significantly improved learning and memory dysfunction, reduced Aβ accumulation, Tau hyperphosphorylation, and downregulated IL-1β and IL-18 expression. This result was consistent with the Aβ1-42 model mice. Sal has anti-inflammatory and anti-pyroptosis effects in neurodegenerative diseases (Wang C. et al., 2017; Zhang X. et al., 2020). Here, we demonstrated that Sal could improve neurodegeneration induced by D-gal/AlCl_3_. Its primary mechanism is inhibiting TLR4/MyD88/NF-κB/NLRP3. All studies were combined, the efficacy of Sal is: (1) that it directly prevents the activation of the NLRP3 inflammasome by suppressing the accumulation of Aβ; and (2), that it downregulated the expression of inflammatory-related proteins, to indirectly inhibit NLRP3 inflammasome-dependent pyroptosis.

To further determine whether Sal inhibited pyroptosis by targeted NLRP3 inflammasome, we established a Nigericin-induced PC12 cell impairment model. It has been reported that Nigericin was an NLRP3 activator, which can reverse Metformin's protective effect and thereby increase the release of pro-inflammatory factors (Zhang J. et al., 2020). Our study agrees with the above studies. In Nigericin and D-gal group, the increased expression level of NLRP3, ASC, and cleaved Caspase-1 was detected in the culture supernatant of PC12 cells, and a corresponding increase in the expression of the NLRP3 downstream proteins cleaved GSDMD, the release of pro-inflammatory factors IL-1β and IL-18 increased, which suggests that Nigericin activated NLRP3 inflammasome. By contrast, the expression of NLRP3 and its downstream proteins was significantly reduced in the Sal group of cells without Nigericin stimulation. Consequently, the above experiment further validated that Sal inhibited pyroptosis by the targeted NLRP3 inflammasome.

In summary, this study expands knowledge on the relationship between NLRP3 inflammasome-mediated pyroptosis and AD. The above data revealed that the efficacy of Sal was similar to DNP, and has a prominent preventive effect in the pathogenesis of AD. It effectively alleviated Aβ aggregation, Tau hyperphosphorylation, neuroinflammation, and pyroptosis. Although DNP displays a neuroprotective effect and is widely used in treating AD, multiple studies have illustrated that it causes adverse effects, including hostility, somnolence, fecal incontinence, nausea, and rhinitis (Adlimoghaddam et al., 2018). Consequently, Sal may be a potential candidate for the treatment of AD through inhibiting the NLRP3 inflammasome-mediated pyroptosis.

## Conclusion

**Figure 11:**
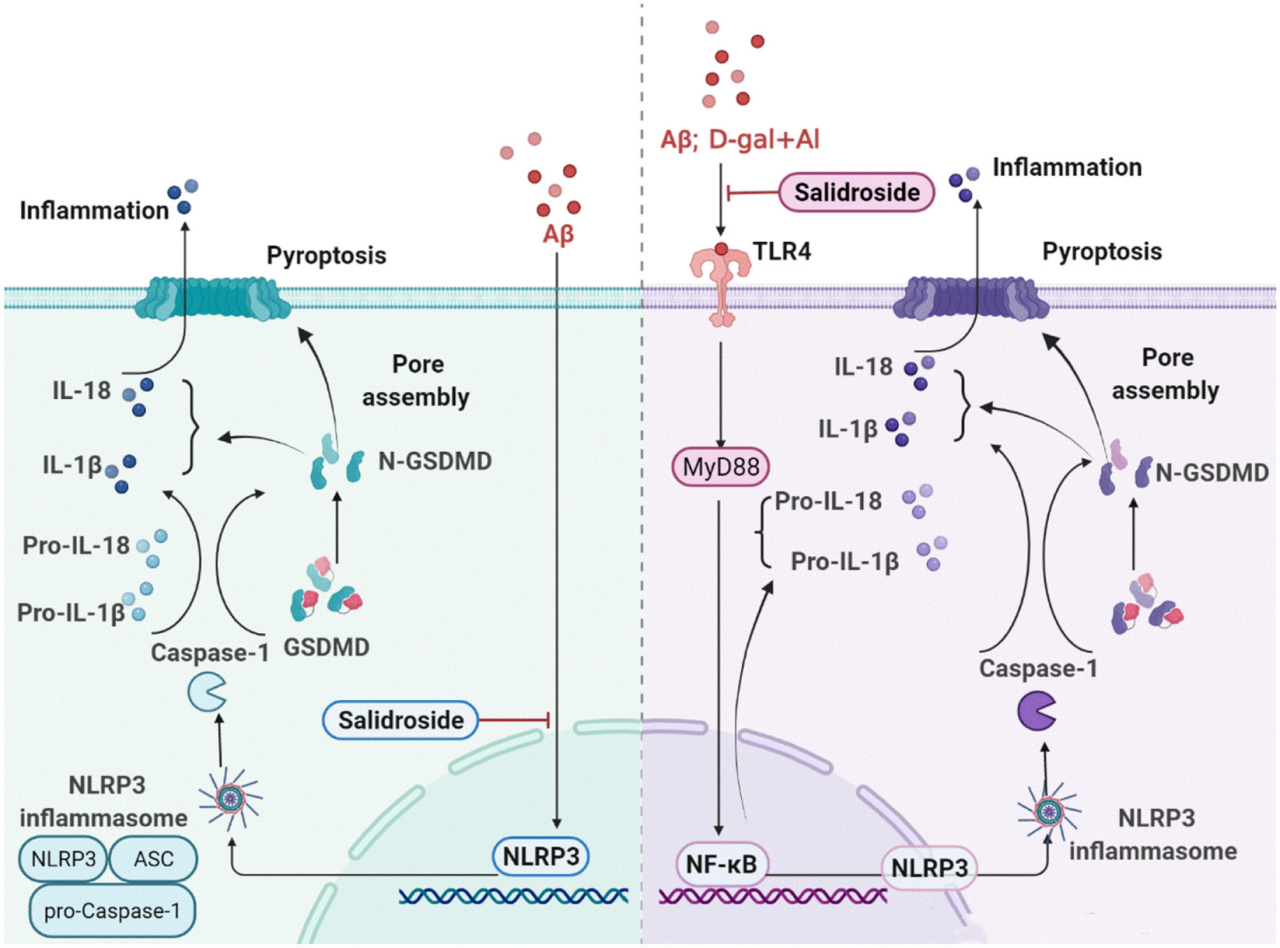
Sal ameliorates AD by suppressing NLRP3-mediated pyroptosis. The primary mechanism is described below: (1) Sal directly reducing the accumulation of Aβ, thus inhibited pyroptosis by blocking the NLRP3 inflammasome activation, mainly through inhibiting NLRP3/caspase-1 signaling pathways; (2) Sal indirectly suppressed pyroptosis through inhibiting TLR4, the mechanism may via TLR4/NF-κB/NLRP3 signaling pathways. These results exhibited that inhibiting pyroptosis through treatment by Sal could be a new approach in the therapeutic treatment of AD.

The present study revealed that activation of NLRP3 inflammasomes is a crucial target for pyroptosis induced by Aβ1-42 and D-gal/AlCl_3_. Thus, if treated with Sal, significant pathological improvement of AD could potentially be achieved by inhibiting the activation of NLRP3 inflammasome and the release of a pro-inflammatory factor downstream of NLRP3 and thus inhibiting pyroptosis. Its potential mechanism may act directly through the inhibited NLRP3/Caspase-1 or indirectly via the suppressed TLR4/NF-κB/NLRP3/Caspase-1 signal pathway. The specific mechanism is shown in Figure 11. These findings provide new insights into the mechanisms underlying the pathogenesis of AD and a unique perspective on the prevention and cure of the disease.

## Data Availability Statement

The original contributions presented in the study are included in the article/Supplementary Material, further inquiries can be directed to the corresponding author/s.

## Ethics Statement

The animal study was reviewed and approved by Ethics Committee of China Pharmaceutical University.

## Author Contributions

YCa conducted most of the experiments, wrote the manuscript, and performed the data analysis and interpretation. YCh, YF, YW, YZ, and XZ participated in parts of the experiments. LZ, TY, and MM guided the design of the study and reviewed the manuscript. All authors contributed to the article and approved the submitted version.

## Conflict of Interest

The authors declare that the research was conducted in the absence of any commercial or financial relationships that could be construed as a potential conflict of interest.

## Publisher's Note

All claims expressed in this article are solely those of the authors and do not necessarily represent those of their affiliated organizations, or those of the publisher, the editors and the reviewers. Any product that may be evaluated in this article, or claim that may be made by its manufacturer, is not guaranteed or endorsed by the publisher.

## Abbreviations

Sal: Salidroside
D-gal: D-galactose
DNP: Donepezil
AlCl_3_: Aluminum chloride
PBS: Phosphate buffer saline
DMSO: Dimethyl sulfoxide
HFIP, 1, 1, 1, 3, 3: 3-Hexafluoro-2-propanol
SDS: Sodium dodecyl sulfate
PVDF: Polyvinylidene fluoride
CCK-8: Cell counting kit-8
IHC: Immunohistochemical
IF: Immunofluorescence
ELISA: Enzyme-linked immunosorbent assay
TLR4: Toll-like receptor 4
NF-κB: Nuclear factor-kappa B
P-NF-κB: Phosphorylation Nuclear factor-kappa B
MyD88: Myeloid differentiation factor 88
NLRP3: Nod-like receptor protein 3
ASC: Apoptosis-associated speck-like protein containing CARD
Caspase-1: Cysteinyl aspartate specific proteinase-1
GSDMD: Gasdermin D
IL-18: Interleukin-18
IL−1β: Interleukin-1β
Aβ: β amyloid
P-Tau: Phosphorylation tau
ANOVA: Analysis of variance.
