# Supplementary material for: Salidroside Ameliorates Alzheimer's Disease by Targeting NLRP3 Inflammasome-Mediated Pyroptosis
Source: Front Aging Neurosci. 2022 Jan 21;13:809433. doi: 10.3389/fnagi.2021.809433 (PMC8814655; doi:10.3389/fnagi.2021.809433)
Supplement: Supplementary file 1 [file Data_Sheet_1.docx]

Supplementary materials：

**Supplementary Table S1. Main materials and reagents**

| **Reagents** | **Source** | **Identifier** |
| --- | --- | --- |
| Salidroside | Meilunbio | MB5843 |
| D-galactose | Aladdin | G100367 |
| AlCl_3_ | Aladdin | A104930 |
| Donepezil | Aladdin | D129948 |
| Nigericin | MCE | HY-127019 |
| [Aβ](https://www.medchemexpress.cn/fludarabine.html)1-42 | Sigma | 107761-42-2 |
| HFIP | Aladdin | H107503 |
| Dimethyl sulfoxide | Sigma | D2650 |
| Phenylmethylsulfonyl fluoride | Aladdin | P105539 |
| RIPA Lysis Buffer  Polyvinylidene fluoride | Beyotime  Millipore | P0013B  IPVH00010 |
| Cell counting kit-8 | Beyotime | C0039 |
| Mouse Aβ1-42(Amyloid Beta 1-42) ELISA Kit | Elabscience | E-EL-M3010 |
| Rat IL-18(Interleukin 18) ELISA Kit | Elabscience | E-EL-R0567c |
| Mouse IL-18(Interleukin 18) ELISA Kit | Elabscience | E-EL-M0730c |
| Rat IL-1β(Interleukin 1 Beta) ELISA Kit | Elabscience | E-EL-R0012c |
| Mouse IL-1β(Interleukin 1 Beta) ELISA Kit | Elabscience | E-EL-M0037c |
| DAPI | Thermo Fisher | R37606 |
| ECL kit | Thermo Fisher | 34580 |

**Supplementary Table S2. List of** **antibodies.**

| **Reagents** | **Source** | **Identifier** |
| --- | --- | --- |
| Mouse monoclonal anti-TLR4 | Santa cruz | sc-293072 |
| Rabbit monoclonal anti-cleaved caspase-1 | Invitrogen | PA5-99390 |
| Rabbit monoclonal anti-ASC | Proteintech | 107761-42-2 |
| Rabbit polyclonal anti-IL-1β  Rabbit polyclonal anti-IL-18 | Proteintech  Proteintech | 16806-1-AP  10663-1-AP |
| Rabbit polyclonal anti-Beta Amyloid | Proteintech | 25524-1-AP |
| Mouse monoclonal anti-Tau | Proteinteche | 66499-1-Ig |
| Rabbit polyclonal anti-NLRP3 | Abcam | ab263899 |
| Rabbit monoclonal anti-Tau (phospho S396) | Abcam | ab109390 |
| Rabbit monoclonal anti-MyD88 | Cell Signaling Technology | #4283 |
| Rabbit polyclonal anti-p-NF-κB p65 | Cell Signaling Technology | #3033 |
| Rabbit monoclonal anti-NF-κB p65 | Cell Signaling Technology | #8242 |
| Rabbit polyclonal anti-cleaved Gasdermin D | Cell Signaling Technology | #10137 |
| Mouse monoclonal anti-β-actin | Cell Signaling Technology | #3700 |
| Anti-rabbit IgG, HRP-linked Antibody | Cell Signaling Technology | #7074 |
| Anti-mouse IgG, HRP-linked Antibody | Cell Signaling Technology | #7076 |
| Anti-mouse IgG (H+L) Alexa Fluor(R) 488 | Cell Signaling Technology | #4408 |
| Thermo marker | Thermo Fisher | 26616 |
| Anti- rabbit IgG (H+L) Alexa Fluor(R) 488 | Thermo Fisher | A11008 |

**Supplementary Table S3. The sequences of the siRNA (Rat)**

| **Product number** | **Product name** | **Serial number** |
| --- | --- | --- |
| siG12910142813 | si-r-Nlrp3_001 | GCTTCAGCCACATGACTTT |
| siG12910142836 | si-r-Nlrp3_002 | CCCTGGGATTTCTCCACAA |
| siG12910142858 | si-r-Nlrp3_003 | GCAATGCCCTTGGAGACAT |
| siB161011044323 | NC-siRNA | GGCTCTAGAAAAGCCTATGC |
